# Disruption of Epithelial Barrier Integrity via Altered GILZ/c-Rel/RACK1 Signaling in Inflammatory Bowel Disease

**DOI:** 10.1093/ecco-jcc/jjae191

**Published:** 2024-12-18

**Authors:** Erica Buoso, Mirco Masi, Roberta Valeria Limosani, Francesca Fagiani, Chiara Oliviero, Giorgia Colombo, Luigi Cari, Marco Gentili, Eleonora Lusenti, Lucrezia Rosati, Federica Pisati, Alessandra Pasini, Marco Vincenzo Lenti, Antonio Di Sabatino, Claire Louise Mobbs, Stefan Przyborski, Simona Ronchetti, Cristina Travelli, Marco Racchi

**Affiliations:** Department of Drug Sciences, University of Pavia, viale Taramelli 12/14, 27100 Pavia, Italy; Department of Pharmacology, Physiology & Biophysics, Boston University Chobanian & Avedisian School of Medicine, 700 Albany St W302 Boston, MA 02215, USA; Department of Drug Sciences, University of Pavia, viale Taramelli 12/14, 27100 Pavia, Italy; University School of Advanced Studies IUSS, Palazzo del Broletto, Piazza della Vittoria 15, 27100 Pavia, Italy; Department of Drug Sciences, University of Pavia, viale Taramelli 12/14, 27100 Pavia, Italy; Translational Neuropathology Unit, Division of Neuroscience, IRCCS San Raffaele Scientific Institute, via Olgettina 60, 20132 Milan, Italy; Department of Drug Sciences, University of Pavia, viale Taramelli 12/14, 27100 Pavia, Italy; Department of Pharmaceutical Sciences, University of Eastern Piedmont, Largo Donegani 2/3, 28100 Novara, Italy; Pharmacology Division, Department of Medicine and Surgery, University of Perugia, Piazzale Gambuli 1, 06132 Perugia, Italy; Pharmacology Division, Department of Medicine and Surgery, University of Perugia, Piazzale Gambuli 1, 06132 Perugia, Italy; Pharmacology Division, Department of Medicine and Surgery, University of Perugia, Piazzale Gambuli 1, 06132 Perugia, Italy; Pharmacology Division, Department of Medicine and Surgery, University of Perugia, Piazzale Gambuli 1, 06132 Perugia, Italy; Cogentech Ltd. Benefit Corporation With a Sole Shareholder, via Adamello 16, 20139 Milan, Italy; Department of Internal Medicine and Medical Therapeutics, University of Pavia, Campus della Salute, presso Policlinico San Matteo, viale Camillo Golgi 19, 27100 Pavia, Italy; Department of Internal Medicine, Fondazione IRCCS Policlinico San Matteo, viale Camillo Golgi 19, 27100 Pavia, Italy; Department of Internal Medicine and Medical Therapeutics, University of Pavia, Campus della Salute, presso Policlinico San Matteo, viale Camillo Golgi 19, 27100 Pavia, Italy; Department of Internal Medicine, Fondazione IRCCS Policlinico San Matteo, viale Camillo Golgi 19, 27100 Pavia, Italy; Department of Internal Medicine and Medical Therapeutics, University of Pavia, Campus della Salute, presso Policlinico San Matteo, viale Camillo Golgi 19, 27100 Pavia, Italy; Department of Internal Medicine, Fondazione IRCCS Policlinico San Matteo, viale Camillo Golgi 19, 27100 Pavia, Italy; Department of Biosciences, Durham University, South Rd, Durham DH1 3LE, UK; Department of Biosciences, Durham University, South Rd, Durham DH1 3LE, UK; Pharmacology Division, Department of Medicine and Surgery, University of Perugia, Piazzale Gambuli 1, 06132 Perugia, Italy; Department of Drug Sciences, University of Pavia, viale Taramelli 12/14, 27100 Pavia, Italy; Department of Drug Sciences, University of Pavia, viale Taramelli 12/14, 27100 Pavia, Italy

**Keywords:** IBD, inflammation, glucocorticoids, intestinal permeability, RACK1, GILZ

## Abstract

**Background and Aims:**

Given the role of Receptor for Activated C Kinase 1 (RACK1) in both immune cell activation and in the maintenance of the intestinal epithelial barrier integrity, we investigated whether it was involved in inflammatory bowel disease (IBD).

**Methods:**

RACK1 expression was analyzed in intestinal mucosal samples of healthy and IBD patients, in mice with chemically induced colitis, and in diseased in vitro 2D and 3D coculture models by luciferase assay, reverse transcription-quantitative PCR, Western blotting, immunofluorescence, and immunohistochemistry. Based on our finding that glucocorticoid-induced leucine zipper (GILZ or *tsc22d3*) positively correlates with RACK1 expression in IBD patients, GILZ knockout mice and cell silencing experiments were performed.

**Results:**

RACK1 was significantly decreased in IBD, especially in ulcerative colitis. This was associated with an NF-κB/c-Rel-related mechanism, correlating with decreased GILZ protein expression. GILZ depletion confirmed a decrease in RACK1 expression, which favored SRC activation and led to a significant reduction in E-cadherin, resulting in impaired epithelial barrier integrity. Finally, our data highlighted that this novel mechanism could be considered to develop new therapies since dexamethasone, the first line of treatment in IBD, restored RACK1 expression through the glucocorticoid receptor in a c-Rel/GILZ-independent manner.

**Conclusions:**

We provide the first evidence that an alteration of RACK1/SRC/E-cadherin regulatory mechanism, correlating with decreased GILZ protein expression, is involved in epithelial barrier disruption. The clinical relevance is based on the fact that this mechanism involving GILZ/c-Rel-related RACK1 expression could be considered to improve IBD therapies, particularly in patients with low or no response to glucocorticoid treatment.

## Graphical Abstract

**Graphical Abstract:**
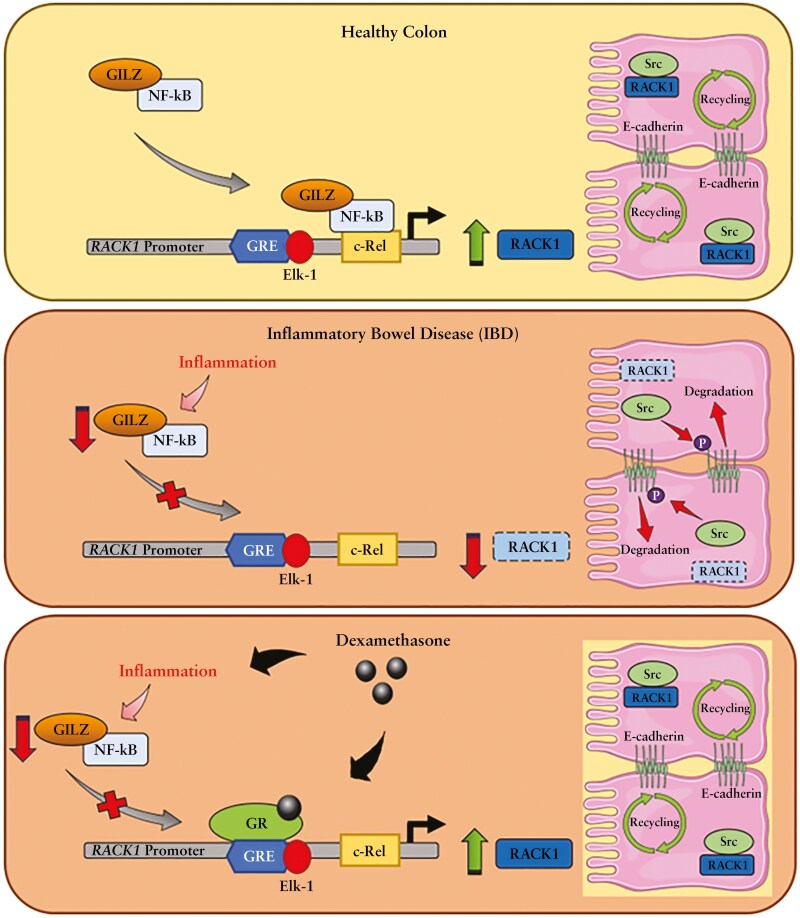

## 1. Introduction

Inflammatory bowel disease (IBD), including Crohn’s disease (CD) and ulcerative colitis (UC), is a group of chronic conditions that affect the gastrointestinal (GI) tract and are characterized by aberrant inflammation of the mucosa and the dysregulation of the immune system.^1^ Although their pathogenesis has not yet been fully elucidated, both CD and UC show a genetic predisposition and present an impaired permeability of the epithelial barrier, gut microbiota abnormalities, and aggravated immune responses.^2,3^ Desmosomes, tight junctions, and adherent junctions are involved in most of the barrier functions and IBD is characterized by a loss of this biological seal due to a primary defect in barrier formation and function as a result of inflammatory processes.^4^ Indeed, tissue-resident macrophages in the inflamed intestine produce high levels of inflammatory cytokines able to disrupt the epithelial barrier,^5^ ultimately aggravating the intestinal inflammation.^6^ The following are the primary mediators produced by these cells: cytokines, including interleukin-1 beta (IL-1β), IL-8, interferon gamma (IFNγ), and tumour necrosis factor-alpha (TNF-α), a pro-inflammatory cytokine which is chronically elevated at both local and systemic levels in IBD patients. Evidence in IBD suggests that TNF-α and other cytokines have profound effects on intestinal epithelial cell (IEC) function, inducing apoptosis, interfering with the expression of proteins involved in the maintenance of intercellular junctions, and modulating epithelial cell integrity and survival.^7^ Despite having raised controversies on their effectiveness due to their intricate and cellular context-dependent anti-inflammatory and immunosuppressive mechanisms^8^ resulting in their association with severe side effects at systemic level,^9^ glucocorticoids (GCs) remain widely utilized as a first-line therapy in IBD. In this regard, recent and increasing evidence showed that dexamethasone treatment of in vivo IBD models resulted in the reduction of colonic concentrations of IL-6, IL-1β, and TNF-α and in the amelioration of macroscopic and microscopic colonic lesions and colonic weight.^10–15^ Among the different proteins regulated by GCs and considering the inflammatory and immune-related features of IBD, a possible important role in this context could be played by the Receptor for Activated C Kinase 1 (RACK1), a scaffold protein involved in different biological events, including neuronal activity,^16,17^ cancer development,^18–20^ and immune response.^21–26^ In the immune context, RACK1 is associated with the activity of Protein Kinase C beta II (PKCβII) and the RACK1-PKCβII complex is strongly correlated with the production of pro-inflammatory cytokines TNF-α and IL-8 and the surface marker CD86.^27^ Due to an androgen/glucocorticoid responsive element (ARE/GRE) half-site in its promoter region,^28,29^ RACK1 is transcriptionally regulated by a complex hormonal balance between androgens and GCs in both the immune^22,30^ and in other contexts.^17,18^ RACK1 has also been recognized as an important molecular component in the intestinal epithelium, where it promotes epithelial cell–cell adhesion,^31^ differentiation, and apoptosis, regulates crypt cell proliferation and regeneration,^32^ and maintains epithelial barrier integrity.^33,34^ Here, we reported that RACK1 is significantly downregulated in both in vitro and in vivo IBD models, consistent with patient data analysis that also showed a significant positive correlation with the GC-induced leucine zipper (GILZ or tsc22d3) protein, playing a putative role in IBD.^35–37^ Consequently, RACK1 transcriptional regulation was investigated in IBD models to dissect its related molecular mechanisms in the intestinal epithelium context. Finally, we investigated whether a clinically relevant anti-inflammatory treatment, dexamethasone, could restore RACK1 expression and epithelial barrier integrity to unravel the GC-related mechanism.

## 2. Materials and Methods

### 2.1. Chemicals, culture media, and supplements

Dexamethasone (PubChem CID: 15159004), Mifepristone (RU486) (PubChem CID: 55245), Phorbol 12-myristate 13-acetate (PMA) (PubChem CID: 27924), BAY 11-7085 (PubChem CID: 5353431), 2,4-Dinitrobenzenesulfonic acid (DNBS) (PubChem CID: 6959), human IFNγ and Lipopolysaccharide (LPS) from *Escherichia coli* serotype 0127:B8, cell culture media and supplements were purchased from Sigma Aldrich (St Louis, MO, USA). Dextran sulfate sodium (DSS) salt (colitis grade, 36 000–50 000 M wt) (PubChem CID: 53788809) was purchased from MP Biomedicals (Solon, OH, USA). The anti-human RACK1 (sc-17754), c-Rel (sc-6955), SRC-1 (sc-32789), ZO-1 (sc-33725), and GAPDH (sc-47724) mouse monoclonal antibodies were purchased from Santa Cruz Biotechnology (Dallas, TX, USA). The mouse monoclonal anti-β-tubulin antibody (T0198) was from Sigma Aldrich (St Louis, MO, USA). The rabbit monoclonal N-cadherin (#13116) and E-cadherin (#3195) antibodies were from Cell Signaling Technology. The mouse monoclonal Lamin A/C (612162) and E-cadherin (#610182) antibodies were purchased by BD Transduction Laboratories (Milan, Italy). Pan-cytokeratin was purchased by Novus Biol NB-600579. The rabbit monoclonal antibody Occludin (ab216327) and rabbit polyclonal antibodies ZO-1 (ab59720) and GILZ (ab197987) were purchased by Abcam. Host-specific peroxidase-conjugated IgG secondary antibodies were purchased from ThermoFisher Scientific (Waltham, MA, USA). Electrophoresis reagents were from Bio-Rad (Richmond, CA, USA). Immunofluorescence (IF) detection of primary antibodies was performed using goat anti-Mouse IgG (H+L) Highly Cross-Adsorbed Secondary Antibody, Alexa Fluor Plus 488 (#A-11029) or 594 (#A-11032) and goat anti-rabbit IgG (H+L) Highly Cross-Adsorbed Secondary Antibody, Alexa Fluor Plus 488 (#A-11034) purchased from ThermoFisher Scientific (Waltham, MA, USA).

### 2.2. Cell cultures and treatments

The human colorectal adenocarcinoma Caco-2 cell line was obtained from the American Type Culture Collection (ATCC TIB-202; Manassas, VA, USA). Human THP-1 cells were purchased from the European Collection of Authenticated Cell Cultures (ECACC, Salisbury, UK). THP1-Blue™ NF-kB cells were derived from the THP-1 (Invitrogen, UK). Human primary fibroblasts were purchased from ThermoFisher Scientific, (Loughborough, UK). Cells were maintained at 37°C in 5% CO_2_ incubator and cultured according to the manufacturer’s instructions. The experiments were carried out on passages 8–18.

BAY 11-7085, dexamethasone, and mifepristone treatments were performed according to literature data^38–41^ and described in more detail in the Supplementary Materials.

### 2.3. Cell viability

Cell viability was assessed by a colorimetric assay based on the reduction of MTT (3-(4,5-dimethylthiazol-2yl)-2,5-diphenyl-tetrazolium bromide) and quantified by reading absorbance at 570 nm wavelength^42^ using Synergy HT multi-detection microplate reader (Bio-Tek, Winooski, VT, USA).

### 2.4. In vitro 2D and 3D coculture models mimicking healthy and diseased state of the human intestine

The 2D coculture and 3D models were developed according to literature data^34,43,44^ and described in more detail in the Supplementary Materials.

### 2.5. Monitoring of barrier integrity by transepithelial electrical resistance

Transepithelial electrical resistance (TEER) was measured using an Ohm-meter to assess the barrier development of the Caco-2 cell layer and monitor its integrity in the presence of inflammatory stimuli^32–34^ in 2D^34^ and 3D models.^44^ Further details are described in the Supplementary Materials.

### 2.6. In vitro 3D model paraffin embedding and hematoxylin and eosin staining

In vitro 3D models were washed in PBS 1× prior to fixation in 4% paraformaldehyde for 2 hours at room temperature (RT). Samples were dehydrated through a series of ethanols, followed by incubation in Histoclear (National Diagnostics, USA) and then in 1:1 Histoclear:wax. Then, models were further incubated in wax before embedding and sectioning according to literature data.^43,44^ Further details are described in the Supplementary Materials.

### 2.7. In vitro 3D model immunofluorescent staining

Paraffin-embedded samples were deparaffinized in Histoclear and rehydrated through 100%, 95%, and 70% ethanol and PBS 1×. Antigen retrieval was performed by incubation in citrate buffer at 95°C for 20 minutes. Samples were blocked in 20% newborn calf serum (Fisher) in 0.4% Triton-X-100 PBS for 1 hour. Primary antibodies, RACK1, Occludin, E-Cadherin (#610182), and ZO-1 were diluted 1:100 in blocking buffer and incubated at 4°C overnight. Slides were washed 3 times in PBS 1×, incubated in Highly Cross-Adsorbed Secondary Antibodies Alexa Fluor diluted 1:1000 in blocking buffer for 1 hour. Then slides were washed again 3 times in PBS 1× before mounting in Vectashield with DAPI (Vector Labs, Peterborough, UK) and imaging on the Zeiss 880 confocal microscope.^44^

### 2.8. Induction of experimental colitis in mouse models

BALB/c and C57BL/6 male mice were housed under specific pathogen-free conditions, 12-hour light/dark cycle at 21 ± 1°C and 50 ± 5% humidity receiving water and food ad libitum, in accordance with the Animals (Scientific Procedures) Act 1986 Amendment Regulations (SI 2012/3039) and the EU Directive 2010/63/EU and in compliance with the ARRIVE guidelines.^45^ Animal care followed the Italian regulations for the protection of animals used for experimental purposes and was approved by the Ministry of Health (120/2018 DB064.27 of 04/10/2017). DNBS and DSS mouse models of experimental colitis were previously obtained and described in our reference.^46^ Briefly, colitis was induced by the administration of DNBS (3 mg in 100 μL of 50% ethanol in 0.9% NaCl solution), which was injected intrarectally with a 0.05-mM catheter inserted 4 cm proximally into the anus. Vehicle alone (100 μL of 50% ethanol) was administered in control experiments (Sham). The animals were observed for 5 days before the sacrifice. Colitis was also induced with the administration of 1.5% DSS in autoclaved drinking water ad libitum for 6 days. The DSS-containing water was substituted on Day 7 and mice were euthanized on Day 9.^46^ RACK1 expression was also studied in our GILZ knockout (KO) mice, which were previously generated and thoroughly characterized.^47^ Finally, the effect of dexamethasone was investigated on our previously obtained and thoroughly characterized DSS-induced colitis mice.^35^

### 2.9. Hematoxylin/eosin, immunohistochemistry, and IF

Hematoxylin/eosin (H&E), immunohistochemistry, and IF were performed and blinded by Cogentech SRL service. To assess histological features H&E (Diapath) staining was performed according to standard protocol and samples were mounted in Eukitt (Bio-Optica). Histological visualization of intestinal mucins was performed using Alcian Blue pH 2.5/PAS staining (Diapath 0100209). For immunophenotypical analysis, paraffin was removed with xylene and the sections were rehydrated in graded alcohol. To expose the target protein, heat-induced epitope retrieval was performed using preheated target retrieval solution for 30 minutes. Following antigen retrieval, the endogenous peroxidase activity was quenched with 3% hydrogen peroxide in distilled water for 10 minutes at RT and the tissue sections were blocked with FBS serum in PBS for 1 hour and incubated overnight with anti-E-cadherin (1:100). The antibody binding was detected using a polymer detection kit (GAM-HRP, Microtech) followed by a diaminobenzidine chromogen reaction (Peroxidase substrate kit, DAB, SK-4100; Vector Lab). All sections were counterstained with Mayer’s hematoxylin and visualized using a bright-field microscope (Leica DM750). IF detection of E-cadherin and RACK1 were performed using goat anti-Mouse (1:100 in PBS 1× for 1 hour) Alexa Fluor Plus 488 and Alexa Fluor Plus 594, respectively. For double IF analysis, paraffin was removed with xylene and the sections were rehydrated in graded alcohol. Antigen retrieval was carried out using preheated target retrieval solution (pH 6.0) for 30 minutes. Tissue sections were blocked with FBS serum in PBS for 60 minutes and incubated overnight with primary antibodies against Pan-cytokeratin (1:100), and RACK1(1:100). Sections were rinsed in PBS and incubated with corresponding secondary antibodies Alexa Fluor 488 or 594 (1:200, Molecular Probes, Invitrogen Life Technologies, Grand Island, NY, USA) for 1 hour at RT. Nuclei were stained with DAPI and the sections were mounted using PBS/Glycerol 50%. IF images were captured using an Olympus Upright BX51 Full Manual microscope.

### 2.10. Data sets, gene expression analysis, and STRING

Expression data from the Gene Expression Omnibus (GEO) database of whole human genome arrays and the ArrayExpress Archive of Functional Genomics Data (ArrayExpress),^48,49^ generated using the Affymetrix Human Genome-U133-Plus-2.0 platform, were downloaded and processed through the Genevestigator V3 suite (NEBION AG, Zurich, Switzerland).^50^

“STRING: functional protein association networks” database (https://string-db.org/cgi/input?sessionId=didPoYPnXmcb&input_page_show_search=on) was used to investigate the interactions involving SRC and 2 of its binding partners, RACK1 and ZO-1.

Data sets, gene expression analysis, and STRING parameters useful for analysis are described in the Supplementary Materials.

### 2.11. Patients

Three colonic biopsies from the most inflamed colonic or ileal mucosal area were collected from 5 patients with CD or UC (overall median age 45 years, interquartile range 38–52, F:M=1.2:1). In all cases, the diagnosis was confirmed by expert physicians, according to the internationally recognized criteria. Biopsies were taken from the most inflamed area (in crescent order, hyperemic mucosa only, erosions, ulcers, punched-out ulcers with spontaneous bleeding). Additionally, 3 biopsies were taken from the rectum in 5 age- and sex-matched healthy controls undergoing colonoscopy for colorectal cancer screening and who turned out to have normal colonic findings. All patients provided written informed consent before the examination; the study protocol was approved by the local ethics committee (protocol number 20140003980).

### 2.12. Reverse transcription-quantitative PCR

Reverse transcription-quantitative PCR (RT-qPCR) was performed on Caco-2 cells or total mouse colon homogenates as previously described.^51,52^ Human and mouse RACK1, GAPDH, and 18S primers were obtained from Qiagen,^17,53^ while human N-cadherin and E-cadherin primers were designed according to literature data.^54^ GAPDH or 18S mRNA was used as an endogenous reference. Transcript quantification was performed with 2^(−ΔΔCT)^ method.

### 2.13. Total homogenates preparation, subcellular fractionation, and IEC isolation

DSS mouse colons or differentiated Caco-2 cell samples were lysed in homogenization buffer according to literature data.^55,56^ To investigate c-Rel level in the nuclear and cytosol fractions, subcellular fractionations were obtained as previously described.^57^ IECs were isolated using a protocol adapted from refs.^58,59^ Protein concentration was determined by the Bradford method (Bio-Rad Laboratories).

### 2.14. Immunoprecipitation

Cytosol fractions of DSS mouse colon or differentiated Caco-2 cell samples were immunoprecipitated as previously described. The immunocomplexes were boiled for 2 minutes after dilution in 2× sample buffer and then centrifuged for 5 minutes at 13 000 *g*.^51,55,56^ The formation of complexes was detected by immunoblotting analysis using specific primary antibodies described in figure legends.

### 2.15. Immunoblotting

The expression of RACK1 (1:1000), β-tubulin (1:1000), E-cadherin (1:1000), N-cadherin (1:1000), SRC-1 (1:250), c-Rel (1:250), GAPDH (1:1000), ZO-1 (1:500), and Lamin A-C (1:1000) was assessed by Western blotting (WB) analysis as described in refs.^20,56^ After WB acquisition, band optical analysis was performed with the ImageJ software (National Institutes of Health, MD, USA). The relative densities of the bands are expressed as arbitrary units and are normalized to the control samples run under the same conditions.

### 2.16. Plasmid DNA preparation, transient transfections, and luciferase assays

The Δ1, Δ7, Δ9, and Δ11 reporter plasmid constructs contain different RACK1 promoter regions as previously described.^28,56^ Dual-Luciferase Reporter Assay System (Promega, Madison, WI, USA) was performed following the manufacturer’s specifications and described in the Supplementary Materials.

### 2.17. Small interference RNA GILZ

Differentiated Caco-2 cells were silenced with siRNA GILZ (sc-43805) or siRNA control (Mission siRNA SIC001) using Lipofectamine RNAiMAX following manufacturer’s instructions (ThermoFisher Scientific). After transfection, cells were analyzed by luciferase assay and immunoblotting.

### 2.18. Enzyme-linked immunosorbent assay determination of TNF-α and IL-8

TNF-α and IL-8 released from PMA-differentiated THP-1 in M1 macrophage cells after coculture treatment were measured in cell-free supernatants obtained by centrifugation at 250 *g* for 5 minutes and processed as previously described.^60^

### 2.19. In vitro 3D model quantification of nuclear NF-κB-induced secreted embryonic alkaline phosphatase

To quantify NF-κB activity in THP1-Blue™ NF-κB macrophages, QUANTI-Blue™ assays (Invivogen, UK) were performed according to the manufacturer’s instructions.^43,44^

### 2.20. Statistical analysis

Data are expressed as means ± standard error of the mean of at least 3 independent experiments. Statistical analyses were performed using GraphPad software, version 7.0. Significant differences were determined using *t* test or the analysis of variance (ANOVA), followed, when significant, by an appropriate post hoc test, as indicated in figure legends. Data sets and gene expression data were analyzed with Kolmogorov–Smirnov normality test to test the distribution of data. *p*-values were calculated using the ordinary 1-way ANOVA (Tukey) test for normally distributed data and the Kruskal–Wallis (Dunn) test for data with skewed distribution. To assess the correlations between gene expression levels, either Pearson’s correlation (for normally distributed data) or Spearman’s correlation (for non-normally distributed data) analysis was used. In all the reported statistical analysis effects were designated as significant if the *p*-value was <0.05.

## 3. Results

### 3.1. RACK1 gene expression in GI tract tissue from healthy and IBD patients

**Figure 1:**
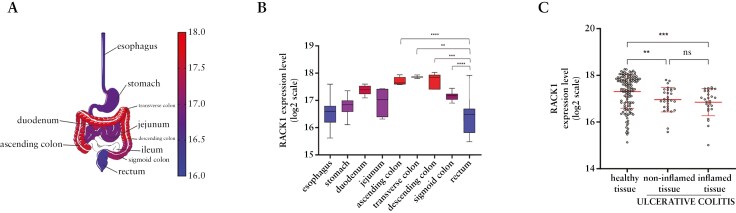
RACK1 expression in GI tract tissue from healthy and UC patients. (A) RACK1 expression levels along the GI tract range from 16 (blue) to 18 (red) on the log_2_ scale. (B) Mean expression levels of RACK1 are shown as a box-and-whiskers plots (Tukey). The scale for the box colors is the same as that for (A). Statistical analysis was performed with Kruskal–Wallis test followed by Dunn’s multiple comparisons test with **p < 0.01; ***p < 0.001; ****p < 0.0001 vs rectum. (C) RACK1 gene expression levels based on microarray data were compared between healthy tissue (*n* = 142), as well as noninflamed (*n* = 30) and inflamed (*n* = 27) tissue from UC patients. The software describes samples obtained from patients with UC as follows: inflamed stands for mucosa biopsy samples derived from the colon from areas with macroscopic and microscopic signs of inflammation, whereas noninflamed stands for mucosa biopsy samples derived from the colon areas without macroscopic and microscopic signs of inflammation. Data are shown by using a scatter plot, with each dot representing a single patient; values are shown in log_2_ scale and expressed as mean ± SD. Statistical analysis was performed with Kruskal–Wallis test followed by Dunn’s multiple comparisons test with *p*-values < 0.05 were considered statistically significant; **p < 0.01, ***p < 0.001, and ns = nonsignificant. Abbreviations: GI, gastrointestinal; UC, ulcerative colitis.

To investigate human RACK1 expression in the GI tract, 284 samples from healthy subjects were analyzed using the Genevestigator V3 suite.^37,50^ RACK1 is expressed at lower levels in the esophagus and rectum, while it is increased in the stomach and jejunum, and reaches its highest expression in the ascending, transverse, and descending colon (Figure 1A) with significant differences compared to the rectum (Figure 1B). Since RACK1 depletion via intestinal-specific inducible KO mice resulted in an inflammatory enterocolitis with human IBD-like features,^33^ a preliminary investigation of RACK1 expression in our UC and CD patient mucosal biopsy samples was performed and showed its significant reduction (Supplementary Figure S1A). RACK1 expression was further analyzed using the GEO and Array Express databases in CD and UC mucosal biopsy samples.^37^ The former showed no significant modulation of RACK1 gene expression (Supplementary Figure S1B), whereas the latter exhibited a significant reduction in both inflamed and noninflamed colon tissues compared to healthy subjects samples (Figure 1C) in accordance with our preliminary patients’ data (Supplementary Figure S1A). These observations on RACK1 decrease support the need to understand the mechanisms that protect barrier function in normal intestine and how the loss of that protection correlates with RACK1 and contributes to IBD pathogenesis.

### 3.2. RACK1 expression is reduced in in vivo and in vitro models of IBD

**Figure 2:**
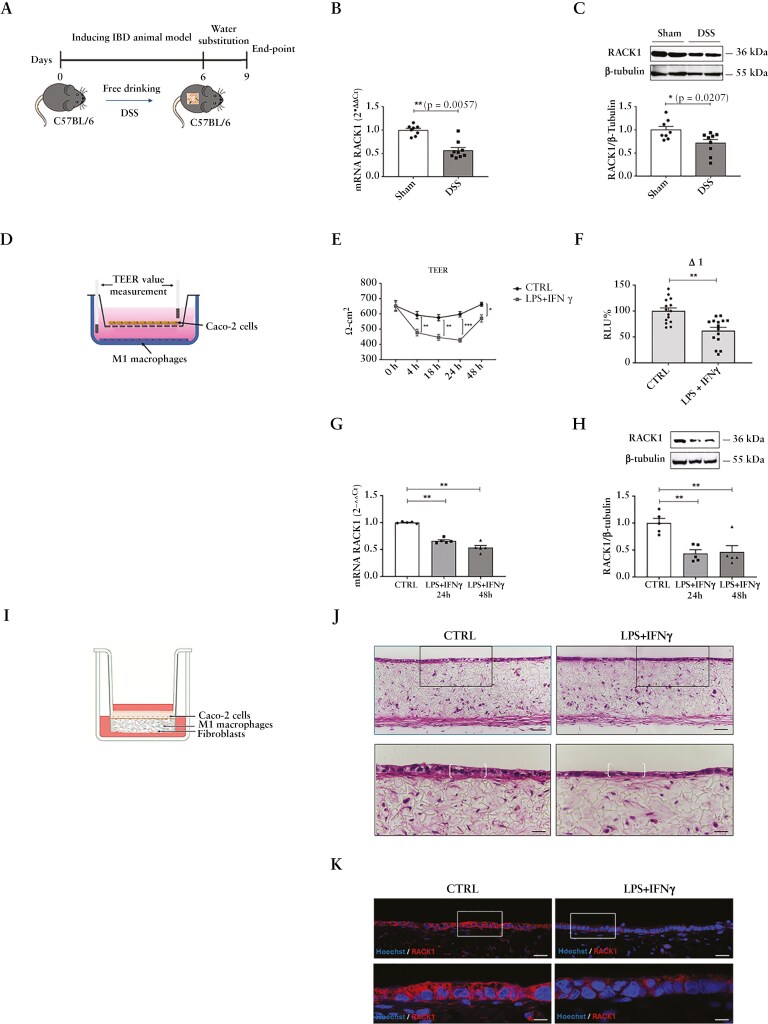
RACK1 expression in in vivo and in vitro models. (A) The figure represents the scheme of DSS-induced colitis. (B) RACK1 mRNA levels were evaluated by qPCR (endogenous reference, GAPDH). (C) RACK1 protein levels were analyzed through WB and normalized to β-tubulin expression. The image is a representative WB. (B, C) Values are means ± SEM and statistical analysis was performed with Student’s *t* test with *p < 0.05. (D) Schematic illustration of the coculture model and TEER measurements. PMA-differentiated THP-1 cells (M1 macrophages) were treated with 10 ng/mL LPS + 10 ng/mL IFNγ for 4 hours before the coculture with IFNγ-primed Caco-2 cells. (E) Caco-2 cell TEER was monitored at selected time points after coculture. Results are expressed as Ω·cm^2^ and corrected for the filter size. Statistical analysis was performed with 2-way ANOVA followed by Bonferroni’s multiple comparison test, with *p < 0.05, **p < 0.01, and ***p < 0.001 vs the corresponding CTRL. (F) 21-day-differentiated Caco-2 cells were transiently transfected with Δ1 RACK1 promoter construct and treated with 10 ng/mL LPS + 10 ng/mL IFNγ for 24 hours. Luciferase activity was expressed as RLU% compared to CTRL, considered as 100%. Each bar represents the mean ± SEM *n* = 5 independent experiments, in triplicate. Statistical analysis was performed with Student’s *t* test, with **p < 0.01. (G, H) 21-day-differentiated Caco-2 cells treated with 10 ng/mL LPS + 10 ng/mL IFNγ for 24 or 48 hours were evaluated for RACK1 mRNA (G) and protein (H) expression. (G) RACK1 mRNA levels were detected by qPCR (endogenous reference, GAPDH). (H) RACK1 protein levels were analyzed through WB and normalized to β-tubulin expression. The image is a representative WB. (G, H) Each value represents the mean ± SEM *n* = 5 independent experiments. Statistical analysis was performed with 1-way ANOVA followed by Dunnett’s multiple comparison test, with **p < 0.01. (I) Schematic illustration of IBD model setup using Alvetex Scaffold technology. (J) H&E images of unstimulated IBD model and 24-hour 10 ng/mL LPS + 10 ng/mL IFNγ stimulated model for comparison. Representative images show histological disease characteristics in stimulated model compared to the control, including altered epithelial architecture, and epithelial thickness, indicated by brackets. Scale bars: 50 μm (upper images), 25 μm (lower images). (K) IF images of RACK1 in unstimulated and in 24-hour 10 ng/mL LPS + 10 ng/mL IFNγ stimulated IBD model. Scale bars 20 μm. Abbreviations: DSS, dextran sulfate sodium; H&E, hematoxylin/eosin; IBD, inflammatory bowel disease; IFNγ, interferon gamma; LPS, lipopolysaccharide; PMA, phorbol 12-myristate 13-acetate; TEER, transepithelial electrical resistance; WB, Western blotting.

To investigate RACK1 expression in IBD, both in vivo and in vitro models were used. Administration of DSS in mice (Figure 2A) causes human UC-like pathologies due to its toxicity to colonic epithelial cells thus resulting in compromised mucosal barrier function^61,62^ as well as impaired body weight and colon length (Supplementary Figure S2A, B). Significantly reduced RACK1 mRNA and protein levels were observed in DSS mice compared to Sham controls (Figure 2B, C). Similar effects were also obtained in whole colons from DNBS mouse (Supplementary Figure S2C–F) thus highlighting RACK1 role in IBD. To specifically assess its downregulation in the epithelium, RACK1 protein expression was analyzed in IECs purified from Sham and DSS mice colons. Our data showed that RACK1 expression is significantly downregulated in this specific intestinal cell type (Supplementary Figure S2G), providing further evidence for the correlation between RACK1 depletion and impaired epithelial barrier integrity. Differentiated Caco-2 cells—a well-established in vitro model of the intestinal epithelial barrier—were characterized according to literature data (Supplementary Figure S3)^54,63,64^ to investigate RACK1 expression. A pro-inflammatory environment was obtained using both a 2D model consisting of differentiated Caco-2 cells cocultured with PMA-differentiated THP-1 in M1 macrophages (Figure 2D–H) and a 3D coculture model representative of the inflamed intestinal mucosa. In the 3D model, a lamina propria-like compartment was created by coculturing fibroblasts and immune cells within and on the surface of the Alvetex scaffold, with an overlying Caco-2 monolayer (Figure 2I–K).^44^ 2D and 3D coculture models were exposed to an inflammatory stimulus consisting of LPS + IFNγ to mimic the diseased intestine in vitro.^32–34,65^ In line with RACK1 role in the immune response,^21–30^ this inflammatory stimulus did not alter its expression in M1 macrophages (Supplementary Figure S4A, B) but induced a significant increase in the release of the pro-inflammatory cytokines TNF-α and IL-8 (Supplementary Figure S4C, D). Moreover, stimulation resulted in significant impairment to the epithelial barrier integrity as evidenced by reduced TEER (Figure 2E). In accordance, a significant reduction of RACK1 promoter activity, mRNA, and protein levels was detected in treated Caco-2 cells (Figure 2F–H), consistent with the pivotal role of RACK1 in epithelial barrier integrity, which was lost in RACK1-silenced cells (Supplementary Figure S3I, J). Similar 2D results were obtained in 3D cocultures mimicking the diseased intestine, showing not only immune cell activation (Supplementary Figure S4E–G), but also epithelial barrier disruption (Supplementary Figure S4H), epithelial thickness, and RACK1 distribution (Figure 2J, K), which was consistent with patient mucosal biopsy samples (Figure 1C; Supplementary Figure S1A). Despite the IBD in vitro models here employed appear to consistently recapitulate our data obtained in DSS mice and from IBD patients, the absence of T cells may limit their IBD-mimicking ability due to: (i) lack of adaptive immune response mediators^7^; (ii) reduced inflammatory signals complexity^66^; (iii) lack of tissue damage and repair dynamics^67^; (iv) limited representation of regulatory mechanisms.^68^ However, despite these limitations suggest that they cannot fully recapitulate the disease, in vitro IBD models still represent a valuable tool to study mechanistic aspects of pharmacological interest and a suitable platform for basic research studies. Therefore, our models are a useful tool to examine RACK1 transcriptional regulation in IBD.

### 3.3. RACK1 expression is positively regulated by c-Rel transcription factor

**Figure 3:**
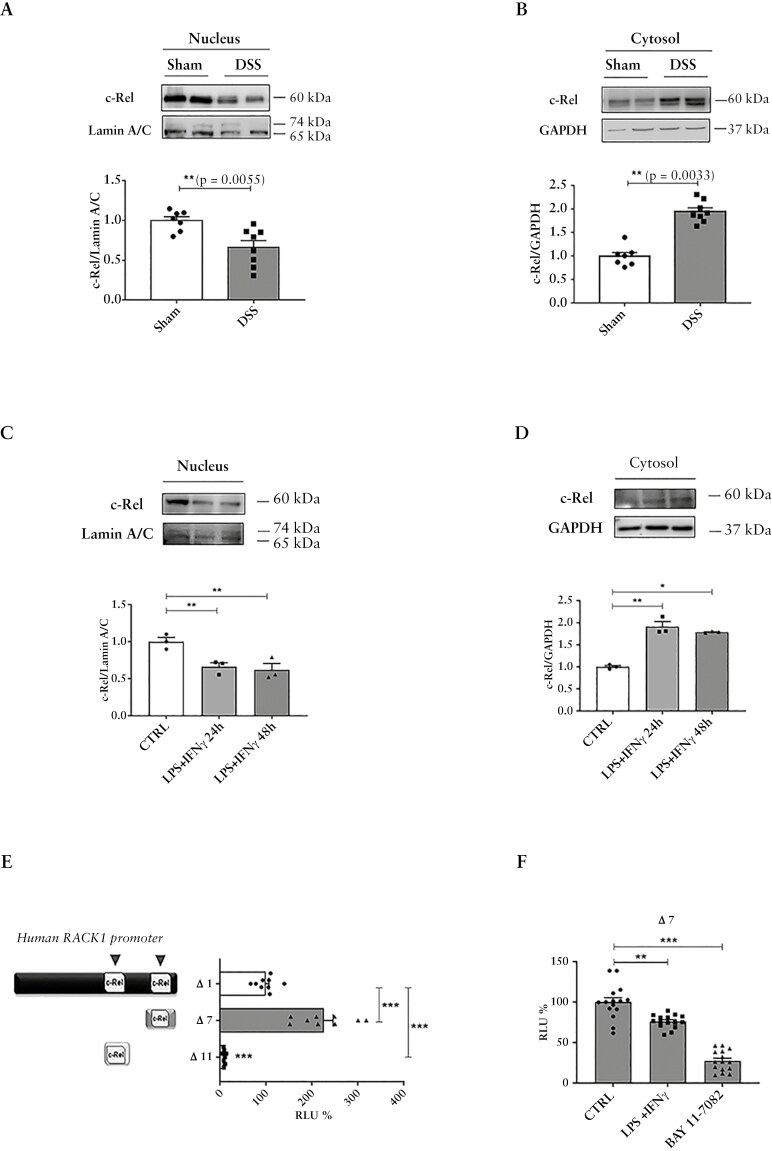
c-Rel involvement in RACK1 transcriptional regulation. (A–D) Analysis of c-Rel expression in nucleus and cytosol fractions of both mice DSS-induced colitis (A, B) and differentiated Caco-2 cells treated with 10 ng/mL LPS + 10 ng/mL IFNγ for 24 or 48 hours (C, D). The images are representative WB and results are shown as c-Rel/lamin A/C ratio for the nuclear fraction (A, C) and as c-Rel/GAPDH ratio for the cytosolic fractions (B, D). (A, B) Each value represents the mean ± SEM and statistical analysis was performed with Student’s *t* test, with **p < 0.01. (C, D) Each value represents the mean ± SEM *n* = 3 independent experiments. Statistical analysis was performed with 1-way ANOVA followed by Dunnett’s multiple comparison test, with *p < 0.05; **p < 0.01. (E) Differentiated Caco-2 cells were transiently transfected with Δ1, Δ7, and Δ11 RACK1 promoter constructs. The basal activity of these luciferase reporter constructs was evaluated through luciferase assay, which was expressed as RLU% and compared to Δ1 values assumed at 100%. Each bar represents the mean ± SEM of *n* = 3 independent experiments, in triplicate. Statistical analysis was performed with 1-way ANOVA followed by Dunnett’s multiple comparison test, with ***p < 0.001. (F) Differentiated Caco-2 cells were transiently transfected with Δ7 and subsequently were treated for 24 hours with 10 ng/mL LPS + 10 ng/mL IFNγ or with 1 μM BAY 11-7085. Luciferase activity was expressed as RLU% and compared to CTRL values assumed at 100%. Each bar represents the mean ± SEM *n* = 5 independent experiments, in triplicate. Statistical analysis was performed with Dunnett’s test, with **p < 0.01, ***p < 0.001. Abbreviations: DSS, dextran sulfate sodium; IFNγ, interferon gamma; LPS, lipopolysaccharide; WB, Western blotting.

c-Rel is one of the canonical, transactivating Rel subunits, with major roles in driving many immunological functions, including the development of regulatory T cells.^69^ c-Rel was also found to be a transcriptional repressor of TNF-α-induced, RelA-dependent, pro-inflammatory gene transcription in ex vivo and in vivo models.^70^ This is in line with its protective effect demonstrated using c-Rel-deficient mice against TNF-α-mediated inflammatory diseases like rheumatoid arthritis^71^ and UC.^72^ In line with this, a significant decrease of c-Rel nuclear translocation was detected in fractionated colons from DSS mice (Figure 3A), which correlated with its increase in the cytosol (Figure 3B), similarly to differentiated Caco-2 cells treated with LPS + IFNγ (Figure 3C, D). Bioinformatic analysis of the human RACK1 promoter revealed the presence of NF-κB/c-Rel transcription factor sites,^28^ similarly to what observed in mouse.^73^ Indeed, functional characterization of RACK1 gene promoter showed that the c-Rel binding sites are important positive regulatory elements for RACK1 expression in different contexts.^20,26,56,73^ Considering that LPS + IFNγ also led to a significant reduction of RACK1 promoter activity, as previously demonstrated by Δ1 construct (Figure 2F), c-Rel role in RACK1 transcriptional regulation was investigated. For this, Caco-2 cells were transiently transfected with 2 deletion mutants (Δ7 and Δ11) of luciferase reporter construct Δ1, which included 2 distinct c-Rel binding sites located inside the RACK1 promoter.^20,56^ Δ7 had an increased basal activity compared to Δ1, indicating that this c-Rel binding site is involved in RACK1 promoter regulation (Figure 3E). Differentiated Caco-2 cells transfected with Δ7 were treated with LPS + IFNγ or with 10 μM BAY 11-7085,^38^ a NF-κB inhibitor. As expected, differentiated Caco-2 cells treated with BAY 11-7085 resulted in a significant decrease of Δ7 luciferase activity, which was also detected in LPS + IFNγ-treated cells (Figure 3F). Altogether, these data support c-Rel involvement in RACK1 transcriptional regulation and indicate that an impaired c-Rel nuclear translocation induced by an inflammatory stimulus results in RACK1 downregulation as previously reported (Figure 2F–H).

### 3.4. Inflammation-induced loss of RACK1/SRC binding negatively impacts the epithelial barrier integrity

**Figure 4:**
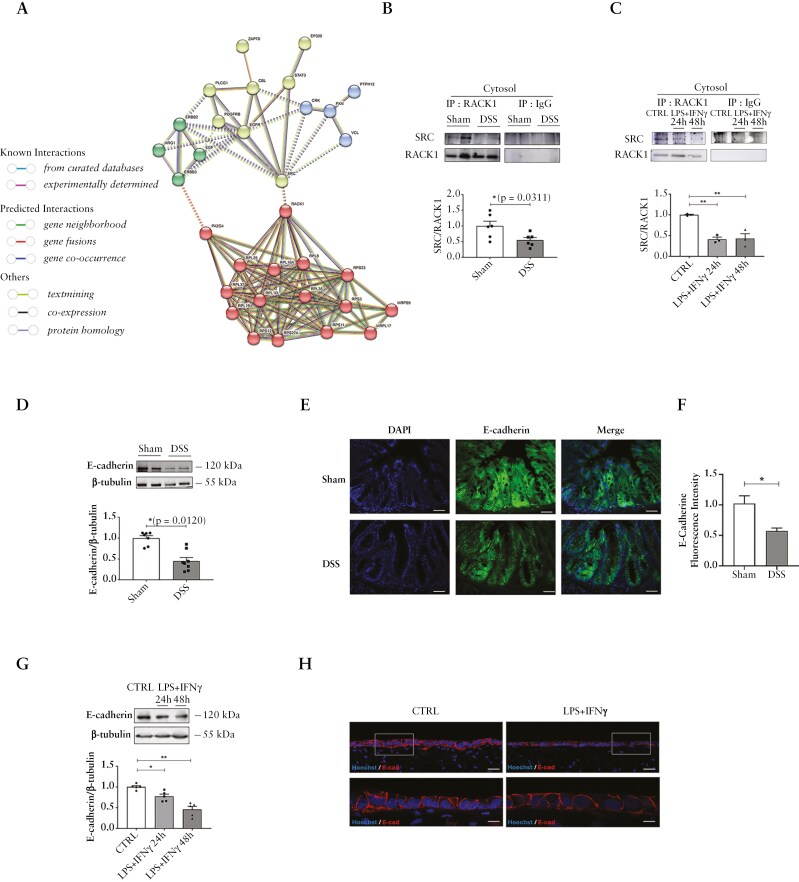
E-cadherin expression is related to RACK1 and SRC complex. (A) SRC and RACK1 interaction. Protein–protein interaction network for *Homo sapiens* SRC was obtained using STRING with full STRING network type. These networks are clustered through *k* means clustering (number of clusters = 4) with a high confidence analysis (STRING interaction score ≥ 0.980). Each cluster is depicted with a different color. The solid and the dotted lines indicate connection within the same and different cluster, respectively. Different color indicates different type of interactions and are shown in the figure. (B, C) Cytosolic fractions of both DSS-induced colitis in mice (B) and Caco-2 cells treated with 10 ng/ml LPS + 10 ng/ml IFNγ for 24 or 48 hours (C) were immunoprecipitated with RACK1 and IgG (Blank) antibody. Immunoprecipitates were analyzed by WB with the anti-SRC and RACK1 antibodies. The images are representative WB. (B) Each value represents the mean ± SEM. Statistical analysis was performed with Student’s *t* test, with *p < 0.05. (C) Each value represents the mean ± SEM *n* = 3 independent experiments. Statistical analysis was performed with 1-way ANOVA followed by Dunnett’s multiple comparison test, with **p < 0.01. (D–G) E-cadherin expression in in vivo and in vitro models. (D) E-cadherin expression in whole colons of mice DSS-induced colitis. The images is a representative WB and results are shown as E-cadherin/β-tubulin ratio. Values are means ± SEM and statistical analysis was performed with Student’s *t* test with *p < 0.05. (E, F) IF images of E-cadherin in distal colon sections of Sham and DSS mouse model and its relative fluorescence intensity. Values are means ± SEM and statistical analysis was performed with Student’s *t* test, with *p < 0.05. Scale bars 25 μm. (G) E-cadherin expression in differentiated Caco-2 cells treated with 10 ng/mL LPS + 10 ng/mL IFNγ for 24 or 48 hours. Each value represents the mean ± SEM *n* = 5 independent experiments. Statistical analysis was performed with 1-way ANOVA followed by Dunnett’s multiple comparison test, with *p < 0.05; **p < 0.01. (H) IF images of E-cadherin in unstimulated and 24-hour 10 ng/mL LPS + 10 ng/mL IFNγ stimulated IBD models. Scale bars 20 μm. Abbreviations: DSS, dextran sulfate sodium; IBD, inflammatory bowel disease; IFNγ, interferon gamma; LPS, lipopolysaccharide; WB, Western blotting.

In healthy colon, RACK1 has been shown to maintain junctional homeostasis of intestinal epithelia by regulating SRC-mediated E-cadherin phosphorylation required for their endocytosis and recycling.^31^ E-cadherin is a calcium-dependent cell–cell adhesion glycoprotein highly expressed in epithelial cells and involved in adherents junction formation.^74,75^ Since RACK1 was found to physically interact with SRC in a full STRING network analysis (Figure 4A), whole colons from DSS mice and Caco-2 cells in in vitro models were examined to evaluate RACK1–SRC binding under disease conditions. A significant decrease in RACK1 binding to SRC was detected in DSS mice and in stimulated cells (Figure 4B, C). Accordingly, a pronounced E-cadherin reduction was detected both in DSS mice and in diseased in vitro models (Figure 4D–H), in line with literature data.^31,76–79^ Furthermore, previous reports indicate that oxidative stress activates SRC and causes its translocation to the membrane, promoting not only the tyrosine phosphorylation of E-cadherin but also occludin and zonula occludens-1 (ZO-1, also known as TJP1).^76,77^ Indeed, ZO-1 physically interacts with SRC in a full STRING network analysis (Supplementary Figure S5A) and is significantly downregulated from Day 1 after DSS colitis induction.^35,80^ In this regard, our analysis using GEO and Array Express databases showed a significant reduction of ZO-1 expression in UC mucosal biopsy samples of inflamed tissues compared to samples of healthy subjects samples (Supplementary Figure S5B) as also observed in in vitro models (Supplementary Figure S5C–E). Indeed, pharmacological restoration of ZO-1 levels has been previously addressed as a possible therapeutic approach for IBD. In this regard, our previous published data showed that treatment with a recombinant GILZ protein (TAT–GILZ) in a DSS mouse model ameliorated the severity of the disease by increasing ZO-1 expression and consequently reducing gut permeability defects.^35^ GILZ is an early-transcribed anti-inflammatory gene^35,81^ that was found significantly reduced in UC mucosal biopsies.^36,37^

### 3.5. GILZ involvement in the regulation of RACK1

**Figure 5:**
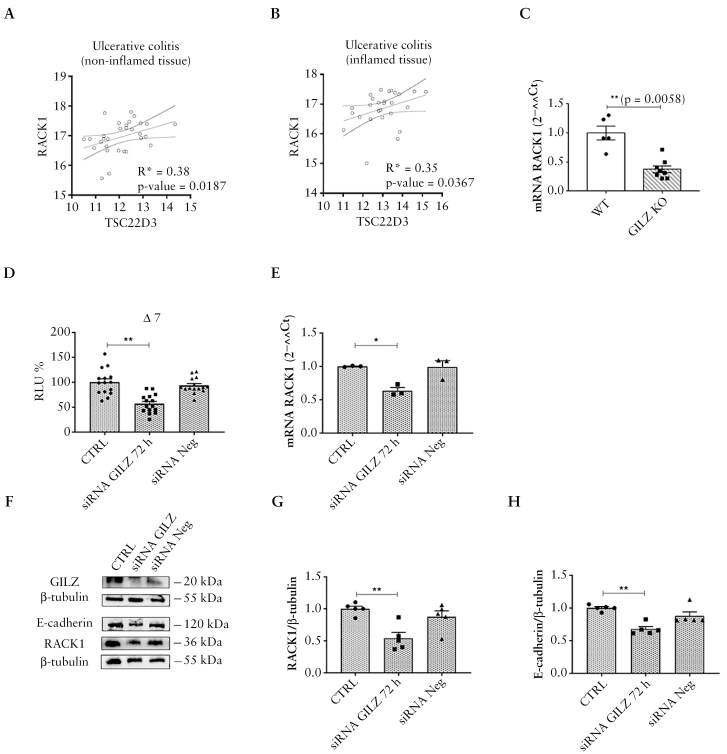
GILZ involvement in RACK1 expression regulation. (A, B) Correlations between the RACK1 and GILZ or tsc22d3 expressions in noninflamed tissue (A), and inflamed tissue (B) from UC patients are shown. Spearman’s correlation analysis was used. (C) RACK1 expression in whole colons of GILZ KO mice were evaluated by qPCR (endogenous reference, 18S). Values are means ± SEM and statistical analysis was performed with Student’s *t* test with **p < 0.01. (D) Differentiated Caco-2 cells (CTRL), with negative siRNA GILZ (siRNA Neg) or silenced with siRNA GILZ were transiently transfected with Δ7. Luciferase activity was expressed as RLU% and compared to CTRL values assumed at 100%. Each bar represents the mean ± SEM *n* = 5 independent experiments, in quadruplicate. Statistical analysis was performed with Dunnett’s test, with **p < 0.01. (E) Analysis of RACK1 mRNA expression in differentiated Caco-2 (CTRL), silenced with siRNA GILZ cells or transfected with the negative siRNA (siRNA Neg) was performed by qPCR (endogenous reference, GAPDH). Each value represents the mean ± SEM *n* = 3 independent experiments. Statistical analysis was performed with 1-way ANOVA followed by Dunnett’s multiple comparison test, with *p < 0.05. (F) The images are representative WB. (G, H) Analysis of RACK1 and E-cadherin expression in differentiated Caco-2 (CTRL), silenced with siRNA GILZ cells or transfected with the negative siRNA (siRNA Neg). Results are shown as RACK1/β-tubulin ratio (G) and E-cadherin/β-tubulin ratio (H). Each value represents the mean ± SEM *n* = 5 independent experiments. Statistical analysis was performed with 1-way ANOVA followed by Dunnett’s multiple comparison test, with **p < 0.01. Abbreviations: GILZ, glucocorticoid-induced leucine zipper; KO, knockout; UC, ulcerative colitis.

Considering that we previously demonstrated the use of TAT–GILZ, as a pharmacological tool to ameliorate symptoms of DSS-induced colitis^35^ and the role of SRC in tight junctions, the existence of a plausible correlation between gene expression levels of GILZ and RACK1 was investigated. In UC mucosal biopsy samples from both inflamed and noninflamed colon tissues, there was a positive correlation value between GILZ (or tsc22d3) and RACK1 genes, indicating the existence of a co-regulation (Figure 5A, B), which was confirmed in whole colons of GILZ KO mice, where a significant decrease in RACK1 expression was found (Figure 5C). Due to the ability of GILZ to bind NF-κB and modulate transcriptional regulation,^82^ we evaluated whether GILZ could regulate RACK1 expression through NF-κB/c-Rel transcription factor. Differentiated Caco-2 cells transfected with Δ7 and silenced for GILZ showed a pronounced decrease of luciferase promoter activity (Figure 5D) which was paralleled by a significant reduction in RACK1 mRNA (Figure 5E) and protein expression (Figure 5F, G), ultimately followed by a reduction in E-cadherin (Figure 5F, H) and ZO-1 (Supplementary Figure S5F). These observations provide the first evidence that loss of GILZ is involved in the decrease of RACK1 expression and consequently in the disruption of intercellular junctions.

### 3.6. Dexamethasone treatment restores RACK1 expression in diseased intestine models

**Figure 6:**
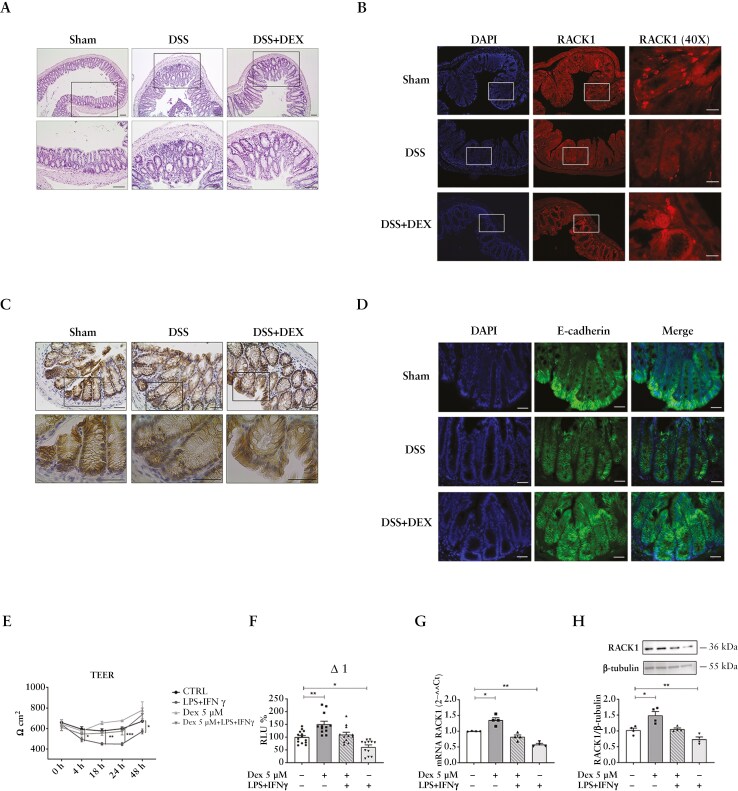
Dexamethasone effect on RACK1 expression. (A) Representative H&E stained images of distal colons of Sham, DSS, and DSS-treated mice with 1 mg/kg of dexamethasone (DSS + Dex). Scale bars: 50 μM. (B, C) IF images of RACK1 (B) E-cadherin (C) in distal colon sections of Sham and DSS and DSS + DEX. Scale bars 25 μm. (D) Immunohistochemistry analysis of E-Cadherin expression in distal colons of Sham, DSS, and DSS + DEX. Scale bars: 25 μm. (E) Dexamethasone antagonizes the reduction of the epithelial barrier integrity induced by LPS + INFγ treatment in 21-day-differentiated Caco-2 cells coculture. Caco-2 cells TEER was monitored at selected time points after coculture. Results are expressed as Ω·cm^2^ and corrected for the filter size. Statistical analysis was performed with 2-way ANOVA followed by Tukey’s multiple comparison test, with *p < 0.05; **p < 0.01, and ***p < 0.001 vs the corresponding CTRL. (F–H) Dexamethasone treatment antagonizes the LPS + IFNγ-induced reduction of RACK1 in 21-day-differentiated Caco-2 cells coculture at promoter (F), mRNA (G), and protein (H) levels. (F) Differentiated Caco-2 cells (CTRL) were transiently transfected with Δ1. Luciferase activity was expressed as RLU% and compared to CTRL values assumed at 100%. Each bar represents the mean ± SEM *n* = 3 independent experiments, in quadruplicate. Statistical analysis was performed with Dunnett’s test, with *p < 0.05 and **p < 0.01. (G) RACK1 mRNA levels were detected by qPCR (endogenous reference, GAPDH). (H) RACK1 protein levels were analyzed through WB and normalized to β-tubulin expression. The image is a representative WB. (G, H) Each value represents the mean ± SEM *n* = 4 independent experiments. Statistical analysis was performed with 1-way ANOVA followed by Dunnett’s multiple comparison test, with *p < 0.05 and **p < 0.01. Abbreviations: DSS, dextran sulfate sodium; H&E, hematoxylin/eosin; IF, immunofluorescence; INFγ, interferon gamma; LPS, lipopolysaccharide; TEER, transepithelial electrical resistance.

GCs are the first-line treatment for IBD due to their ability to promote complete remission, though they cause several undesirable side effects. GILZ and RACK1 are known to be GC-regulated genes.^21,81,83^ RACK1 expression has been demonstrated to be negatively or positively regulated by GCs in a cellular context-dependent manner.^16–30^ Given that dexamethasone resulted in the amelioration of colonic lesions and a reduction in pro-inflammatory cytokine concentration in the DSS mice intestine,^10–15^ it was hypothesized that this treatment could restore the inflammation-induced loss of RACK1 and E-cadherin expression. DSS mice treated with 1 mg/kg of dexamethasone^35^ exhibited improved mucosal integrity, reduced crypt loss (Figure 6A), mucins redistribution (Supplementary Figure S6A), and tissue-specific rescue of RACK1 (Figure 6B) and E-cadherin (Figure 6C, D). In addition, co-immunostaining of RACK1 with the epithelial marker pan-cytokeratin (Supplementary Figure S6B) further confirmed the effect of dexamethasone on RACK1 rescue. To investigate RACK1 transcriptional regulation, differentiated Caco-2 cells were treated with different dexamethasone concentrations selected based on previous data.^28,30,39,40^ These concentrations did not affect the Caco-2 cell viability (Supplementary Figure S6C, D), while a significant upregulation in RACK1 expression at both mRNA and protein levels was observed with 5 µM dexamethasone (Supplementary Figure S6E, F). Thus, 5 µM was selected for subsequent investigations. In line with previous reports,^21,27^ a significant reduction in RACK1 expression was observed in dexamethasone-treated cocultured M1 macrophages (Supplementary Figure S6G), leading to a decrease in the release of TNF-α and IL-8 in the inflamed condition (Supplementary Figure S6H, I). Accordingly, dexamethasone treatment attenuated the epithelial barrier disruption induced by the inflammatory stimulus, restoring TEER levels (Figure 6E) by also reestablishing RACK1 promoter activity, mRNA, and protein expression in differentiated Caco-2 in vitro model (Figure 6F–H). Thus, RACK1 emerges as an interesting target and a potential in vitro pharmacological screening tool in IBD due to its simultaneous and opposing roles in immune and intestinal cells, as evidenced by these dexamethasone data.

Finally, to dissect the dexamethasone mechanism of action on RACK1 expression, luciferase promoter analysis was performed. Based on the presence of a GRE in RACK1 promoter (Supplementary Figure S7A),^28^ differentiated Caco-2 cells were transfected with Δ1 and treated with dexamethasone in the presence or absence of mifepristone, a glucocorticoid receptor (GR) antagonist. Dexamethasone induced a significant increase in RACK1 promoter luciferase activity, which was prevented by 1 µM mifepristone treatment (Supplementary Figure S7B) thus highlighting that dexamethasone acts through GR. This result was further confirmed using Δ9, containing only the GRE site (Supplementary Figure S7A, C).^18,30^ Furthermore, we demonstrated that dexamethasone restored RACK1 expression in a c-Rel/GILZ-independent manner, as cells transfected with Δ7 and treated with dexamethasone in the presence of the inflammatory stimulus did not show restoration of RACK1 promoter activity (Supplementary Figure S7D). These data were consistent with the impaired c-Rel nuclear translocation (Supplementary Figure S7E) and its significant presence in the cytosol (Supplementary Figure S7F). In this context, the novel mechanism of GILZ/c-Rel on RACK1 expression could be considered in patients with low or no response to GC therapy. Indeed, we found that pediatric GC nonresponders were characterized by lower expression of RACK1 (Supplementary Figure S7G), thus paving the way for further investigations.

## 4. Discussion

RACK1 is involved in a plethora of key biological events.^16,19,29^ It has been identified as the scaffold protein for different isoforms of the activated PKC, and the RACK1–PKCβII complex plays an important role in the innate immune response.^21–30^ Given that RACK1 is also involved in maintaining the integrity of the intestinal epithelial barrier,^33^ we first evaluate its expression in GI tissues from healthy and IBD patients, who exhibited a deficiency in RACK1 expression, which was significant UC, whereas in quiescent UC patients, specifically in IECs, RACK1 expression was found upregulated. However, these findings are from a previous limited study conducted of 10 biopsies and require further validation.^59^ Moreover, in accordance with our analysis in UC patients, DSS mouse models and our in vitro 2D and 3D cell cocultures showed a significant RACK1 downregulation that correlated with a relevant decrease in RACK1 binding to SRC, leading to a decrease in E-cadherin expression and ultimately in epithelial barrier disruption. Indeed, E-cadherin depletion in the mouse colon results in a severe enterocolitis phenotype, body weight reduction, and macroscopic and microscopic colonic lesions^84^ as also observed in RACK1-depleted mice.^33^ Moreover, the reduced E-cadherin surface expression induced by IFNγ in colon epithelial cells^85^ and the aggravated DSS-induced colitis phenotype due to E-cadherin deficiency^78^ confirm their role in the maintenance of the intestinal epithelial architecture and their central role in IBD pathophysiology.^86,87^ Furthermore, we provide the first evidence that RACK1 expression positively correlated with GILZ in UC patient mucosal biopsy samples, in line with the decreased GILZ expression demonstrated in our models.^35,37,61,81^ GILZ depletion both in vivo and in vitro significantly affected RACK1 expression, through a c-Rel-related mechanism, consistent with the observed impairment of c-Rel nuclear translocation in our models. This observed GILZ-related RACK1 decrease ultimately correlated with a significant reduction in E-cadherin expression. Moreover, reduced crypt cell differentiation into goblet cell induced by E-cadherin,^84^ GILZ,^37,81^ and RACK1 deficiencies^32^ provides further evidence for the existence of a mechanism with a positive feedback loop involving GILZ and RACK1–SRC complex for epithelial barrier integrity. These results are consistent with UC patients and our previous data showing a significant reduction in ZO-1 expression in DSS colitis-induced mice^35^ due to RACK1 deficiency-related SRC activation^76,77^ that was restored by a TAT–GILZ pharmacological strategy.^35^ Therefore, our data highlight that RACK1-related GILZ expression is critical for sequestering SRC and preventing epithelial barrier dysfunction in colitis, thus contributing to the understanding of how to improve therapies. In this regard, to delve deeper into the complexities of UC, we maintain that future work should consider organoids and patient monocyte-derived macrophages.

**Figure 7:**
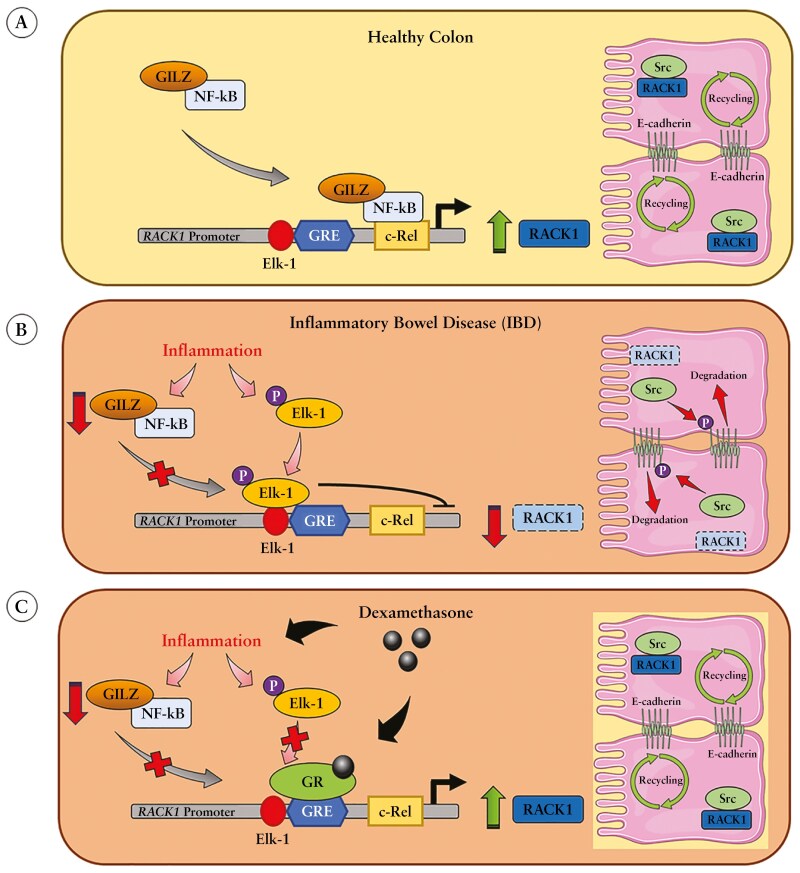
Proposed mechanism of epithelial barrier function-related GILZ/NF-κB (c-Rel)/Elk-1/GR interplay in RACK1 transcriptional regulation. Our current hypothesis in healthy colonic conditions suggests that (A) GILZ/c-Rel (NF-κB) complex plays a positive role in regulating RACK1 transcription, allowing a correct E-cadherin recycling via RACK1-mediated SRC sequestration. Conversely, (B) in IBD, inflammation impairs GILZ/c-Rel nuclear translocation, making Elk-1 binding site accessible for the phosphorylated colonic repressor and transcription factor Elk-1, which may result in loss of RACK1 and SRC sequestration thus increasing E-cadherin degradation. However, (C) IBD treatment with dexamethasone can induce RACK1 upregulation thus restoring the epithelial barrier integrity and function. Abbreviations: GILZ, glucocorticoid-induced leucine zipper; IBD, inflammatory bowel disease.

Since GILZ and RACK1 are GC-regulated genes,^21,81,83^ as a proof of concept, we investigated dexamethasone effect, known to ameliorate the severity of the disease in DSS mice intestine. Dexamethasone restored RACK1 expression through GR binding to its promoter in a GILZ/c-Rel-independent manner, as supported by the observed impairment of c-Rel nuclear translocation in inflamed intestinal cells, in line with dexamethasone literature data.^88^ These results show that this GC-independent mechanism could be exploited by administering TAT–GILZ treatment, as not all of its target genes and downstream effects have been shown to overlap with GC-related effects.^35,89,90^ Therefore, the originality of this work is based on the finding that the mechanism of IBD epithelial barrier dysfunction is related to GILZ-dependent RACK1 downregulation, which ultimately leads to ZO-1 and E-cadherin degradation through SRC activation. Considering these results and existing literature, we hypothesize that the loss of GILZ and the decline in c-Rel-related nuclear translocation could make the Elk-1 binding site, located near the GRE site, more accessible,^28^ thereby repressing RACK1 promoter (Figure 7A, B). The transcription factor Elk-1 acts as a repressor in colonic tissues of DSS mice, as evidenced by a significant increase in its phosphorylation.^91^ Indeed, the suppression of MAPK/ERK/JNK/p38 MAPK signaling inhibited the phosphorylation of Elk-1, which was demonstrated to be beneficial due to the decrease in inflammatory mediators and in the prevention of DSS-induced UC.^91^ Therefore, we propose that GR binding to the RACK1 promoter following dexamethasone treatment could counteract the phospho-Elk-1 effect, thus acting either as a dominant-positive regulator or promoting Elk-1 detachment from the promoter (Figure 7C). Finally, the clinical relevance of this work is based on the fact that although RACK1 pharmacological targeting with GCs may be a suitable therapeutic strategy in IBD, particularly in patients with low or no response to GC therapy,^92^ the novel mechanism of GILZ on RACK1 expression could be considered to improve IBD therapies. In this context, TAT–GILZ, acting by a different mechanism than GCs and successfully described in preclinical studies as a pharmacological tool for the treatment of inflammation or autoimmune diseases,^93–97^ paves the way for the investigation of a new therapeutic strategy.

## Supplementary Material

jjae191_suppl_Supplementary_Figures

## Data Availability

Data available on request.
