# Supplementary material for: Disruption of Epithelial Barrier Integrity via Altered GILZ/c-Rel/RACK1 Signaling in Inflammatory Bowel Disease
Source: J Crohns Colitis. 2024 Dec 18;19(1):jjae191. doi: 10.1093/ecco-jcc/jjae191 (PMC12187379; doi:10.1093/ecco-jcc/jjae191)
Supplement: jjae191_suppl_Supplementary_Figures [file jjae191_suppl_supplementary_figures.docx]

**SUPPLEMENTARY MATERIALS**

**Disruption of epithelial barrier integrity via altered GILZ/c-Rel/RACK1 signaling in inflammatory bowel disease**

Erica Buoso^1,2^ *^#^, Mirco Masi^1,3^ *, Roberta V. Limosani^1^, Francesca Fagiani^4^, Chiara Oliviero^1^, Giorgia Colombo^5^, Luigi Cari^6^, Marco Gentili^6^, Eleonora Lusenti^6^, Lucrezia Rosati^6^, Federica Pisati^7^, Alessandra Pasini^8,9^, Marco Vincenzo Lenti^8,9^, Antonio Di Sabatino^8,9^, Claire Louise Mobbs^9^, Stefan Przyborski^10^, Simona Ronchetti^6^, Cristina Travelli^1^ and Marco Racchi^1^.

**Affiliations**

^1^ Department of Drug Sciences, University of Pavia, Viale Taramelli 12/14, 27100 Pavia, Italy.

^2^ Department of Pharmacology and Experimental Therapeutics, Boston University Chobanian & Avedisian School of Medicine, Boston, MA 02118, USA.

^3^ University School of Advanced Studies IUSS, Piazza della Vittoria 15, 27100 Pavia, Italy.

^4^Translational Neuropathology Unit, Division of Neuroscience, IRCCS San Raffaele Scientific Institute, 20132 Milan, Italy.

^5^ Department of Pharmaceutical Sciences, University of Eastern Piedmont, via Bovio 6, 28100 Novara, Italy.

^6^ Pharmacology Division, Department of Medicine and Surgery, University of Perugia, P.le L. Severi 1, 06132 Perugia, Italy.

^7^ Cogentech Ltd. Benefit Corporation With a Sole Shareholder, Via Adamello 16, Milan, 20139, Italy.

^8^Department of Internal Medicine and Medical Therapeutics, University of Pavia, Viale Golgi 19, 27100 Pavia, Italy;

^9^Department of Internal Medicine, Fondazione IRCCS Policlinico San Matteo, Viale Golgi 19, 27100 Pavia, Italy

^10^Department of Biosciences, Durham University, Durham, United Kingdom.

* These authors have contributed equally to this work

# Corresponding author:

**SUPPLEMENTARY FIGURES**

**
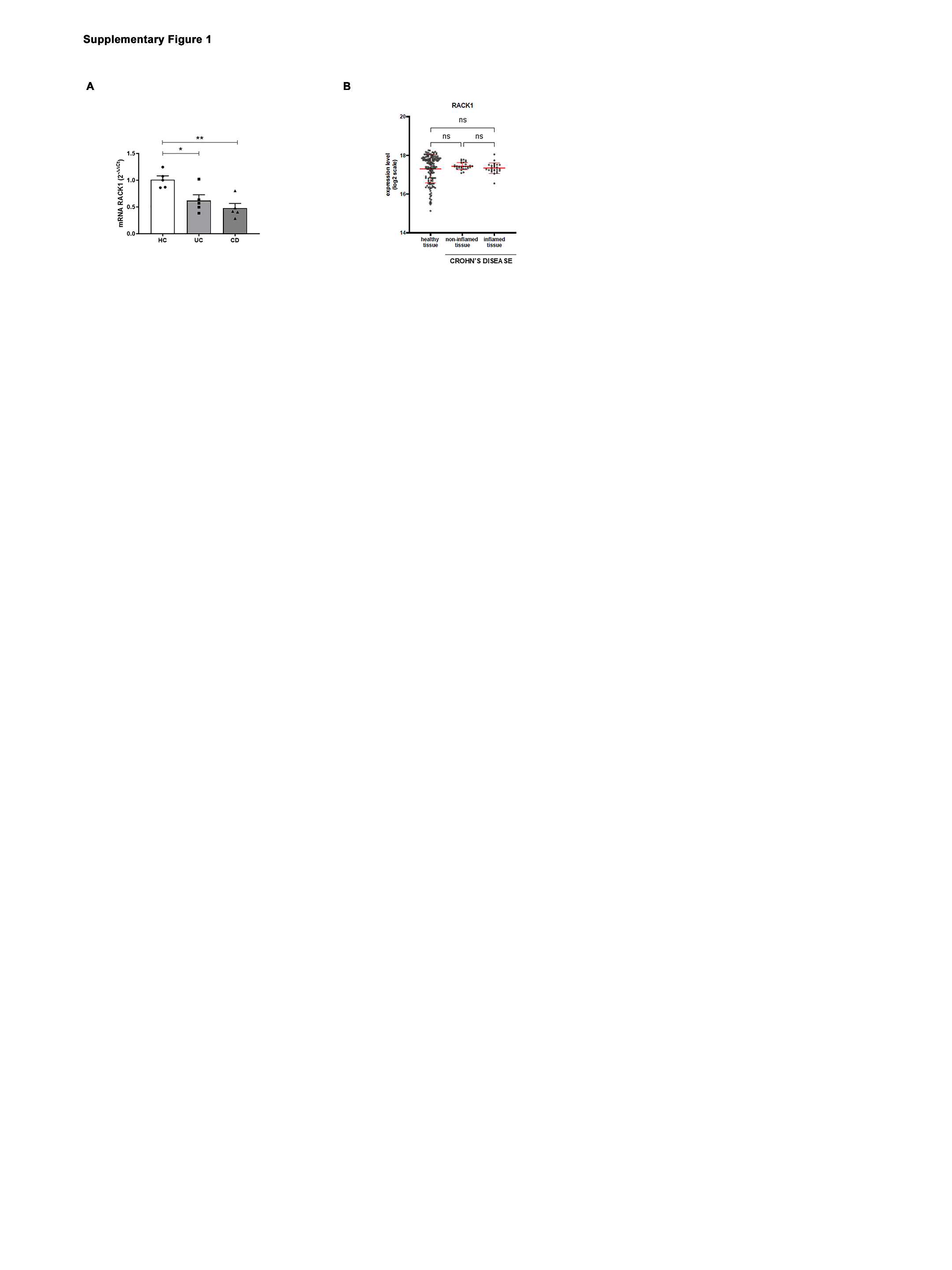
**

**Fig. S1. Evaluation of RACK1 expression in human biopsies from UC and CD patients.**

**A.** RACK1 mRNA levels are reduced in biopsies samples of UC and CD patients compared to healthy colon (HC) controls. Evaluation of RACK1 mRNA was performed by qPCR and normalized to GAPDH mRNA expression. Each value represents the mean ± SEM n = 5 individual samples. Statistical analysis was performed with one-way ANOVA followed by Dunnett’s multiple comparison test, with *p < 0.05 and **p < 0.01. **B.** RACK1 gene expression levels based on microarray data were compared between healthy tissue (n = 142), as well as non-inflamed (n = 29) and inflamed (n = 24) tissue from CD patients. Data are shown by using a scatter plot, with each dot representing a single patient; values are shown in log_2_ scale and expressed as mean ± SD. Statistical analysis was performed with Kruskal-Wallis test followed by Dunn's multiple comparisons test with p-values <0.05 were considered statistically significant; ns = non-significant.

**
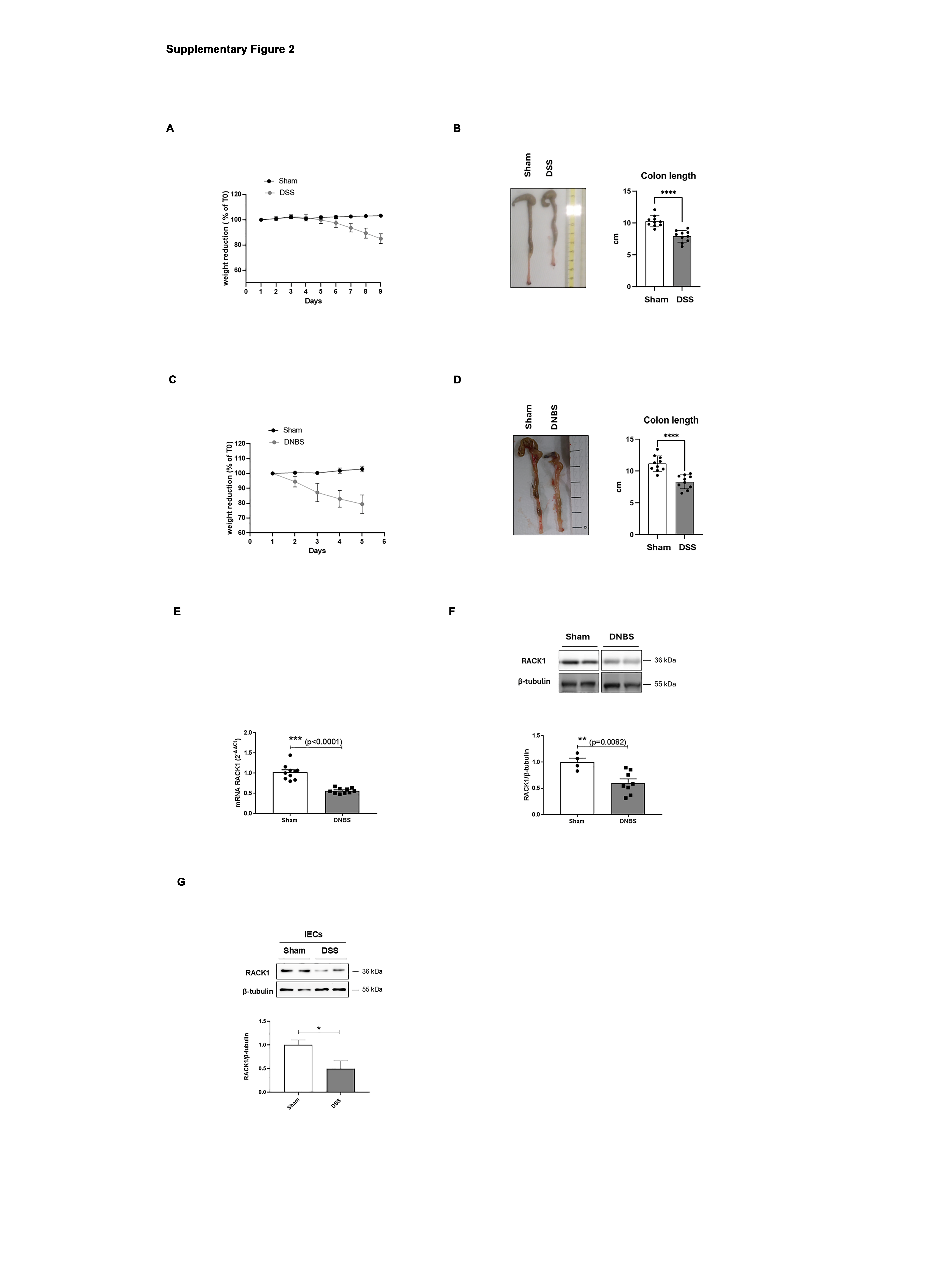
**

**Fig. S2. Evaluation of RACK1 expression in an IBD *in vivo* model**

**A-D.** Analysis of body weight, representative image of colons and colon length of DSS (A, B) and DNBS mice (C, D). Statistical analysis was performed Student’s t-test, with ****p < 0.0001. **E, F.** RACK1 expression is reduced in colon total extracts of DNBS mouse IBD model. **E.** Evaluation of RACK1 mRNA in Sham vs DNBS was performed by qPCR and normalized to GAPDH mRNA expression. **F.** RACK1 protein levels in Sham vs DNBS were analyzed through Western blot and normalized to β­tubulin expression. The image is a representative Western blot. **E, F.** Values are means ± SEM and statistical analysis was performed with Student’s t-test, with **p < 0.01 and ***p < 0.001. **G.** RACK1 protein levels in IECs purified from Sham and DSS mice colons were analyzed through Western blot and normalized to β­tubulin expression. The image is a representative Western blot. Values are means ± SEM and statistical analysis was performed with Student’s t-test, with *p < 0.05.


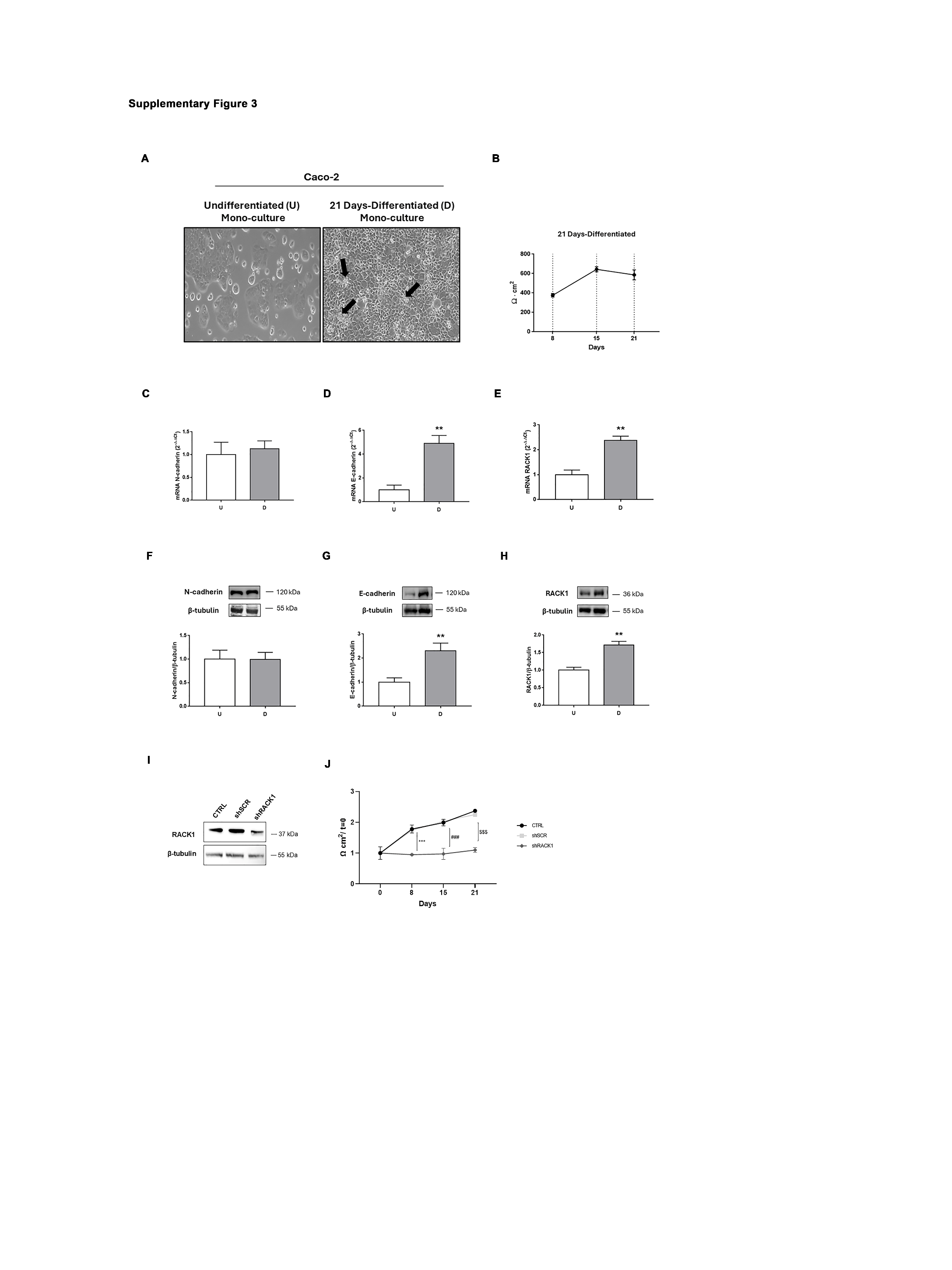


**Fig. S3. Characterization of 21-days-differentiated Caco-2 mono-culture.**

**A.** Phase Contrast Optical Microscope images at 10X magnification of undifferentiated (U) (left panel) and 21-days-differentiated (D) (right panel) Caco-2 cells mono-culture. Black arrows indicate dome formation. **B.** 21-days-differentiated Caco-2 cells TEER was monitored at selected time-points. Results are expressed as Ω·cm2 and corrected for the filter size. **C-H.** Evaluation of N-cadherin, E-cadherin and RACK1 differential expression in U and D Caco-2 cells. **C-E.** N-cadherin (C), E-cadherin (D) and RACK1 (E) mRNA expression analysis was performed by qPCR. Results are normalized to GAPDH mRNA levels. **F-H.** N-cadherin (F), E-cadherin (G) and RACK1 (H) protein levels were analyzed through Western blot and normalized to β-tubulin expression. Images are representative WESTERN BLOT. **C-H.** Each value represents the mean ± SEM n = 4 independent experiments. Statistical analysis was performed with Student’s t-test, with **p < 0.01. **I.** Representative WESTERN BLOT image of RACK1 protein levels in CTRL, shScramble (shSCR) and RACK1-silenced cells (shRACK1) cells. **J.** 21-days-differentiated Caco-2 cells (CTRL and shSCR) wild-type and shRACK1 cells TEER was monitored at selected time-points. Results are expressed as Ω·cm2, corrected for the filter size and normalized to t=0, which corresponds to day 1. Statistical analysis was performed with two-way ANOVA followed by Tukey's multiple comparison test, with ***p < 0.001 vs CTRL (8 Days); ^###^p < 0.001 CTRL (15 Days) and ^§§§^p <0.001 vs CTRL (21 Days).

**
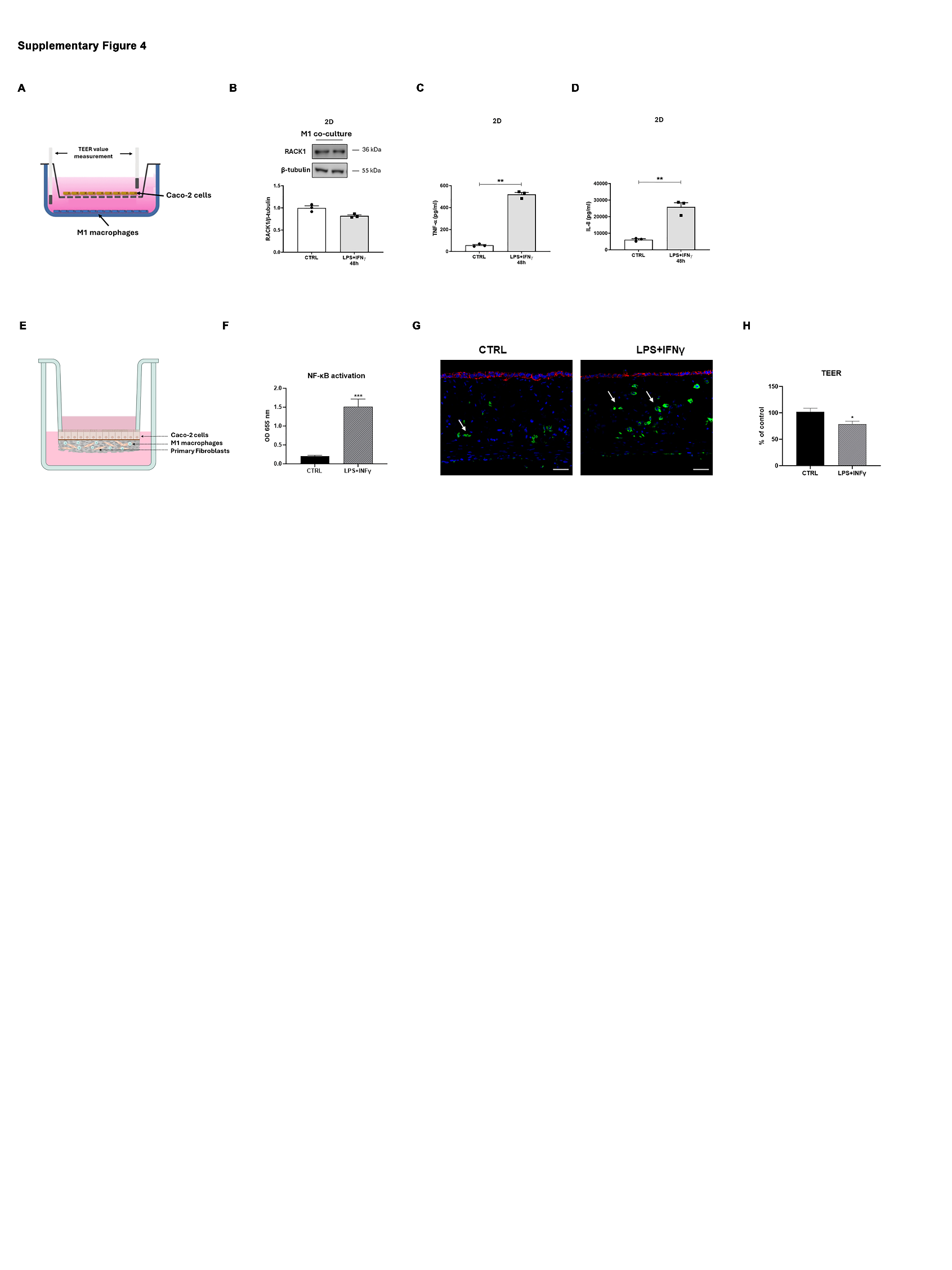
**

**Fig. S4. Functional characterization of 2D and 3D co-culture models.**

**A.** Schematic illustration of the co-culture model and TEER measurements. **B.** RACK1 protein levels in M1 co-cultured macrophages were analyzed through Western blot and normalized to β-tubulin expression. The image is a representative Western blot and values are means ± SEM n= 3 independent experiments. **C-D.** LPS+IFNγ treatment increases TNF-α and IL-8 release in M1 macrophages. After co-culture (as detailed in Materials and Methods section), secretion of cytokines TNF-α (A) and IL-8 (B) was evaluated in cell-free supernatants by sandwich ELISAs. Each value represents the mean ± SEM n = 3 independent experiments. Statistical analysis was performed with Student’s t-test, with **p < 0.01. **E.** Schematic illustration of IBD model setup using Alvetex^®^ Scaffold technology. **F.** 24h 10 ng/mL LPS+10 ng/mL IFNγ stimulation of IBD model increases NF-κB activation compared to control. n=3. *** p< 0.0001. **G.** IF images of immune marker CD14 (green) and epithelial marker E-cadherin (red) in unstimulated and 24h 10 ng/mL LPS+10 ng/mL IFNγ stimulated IBD models. Scale bars 20 μm. **H.** TEER was monitoring in unstimulated and 24h 10 ng/mL LPS+10 ng/mL IFNγ stimulated IBD models (n = 3). Results are expressed as percentage of control.


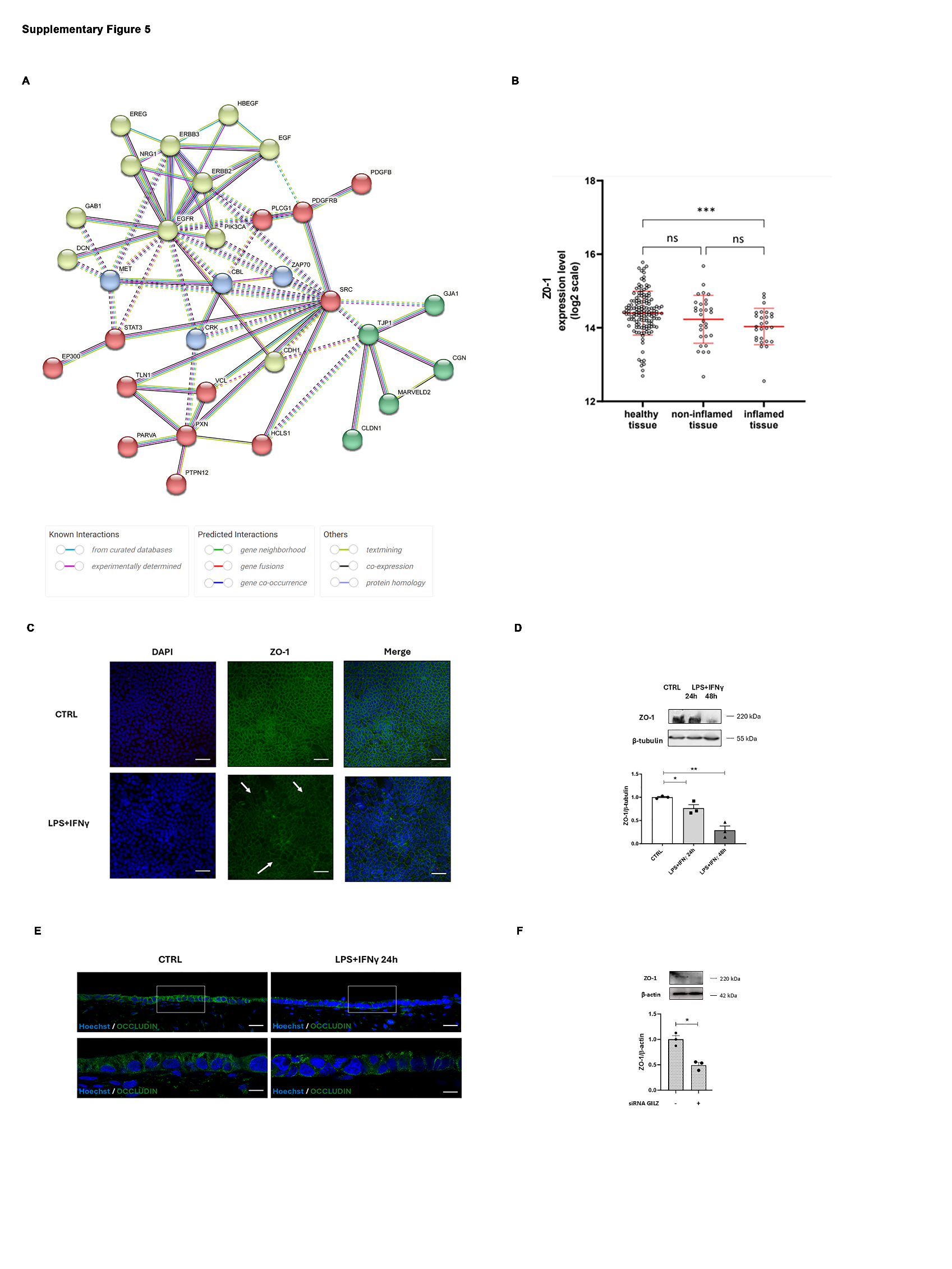


**Fig. S5. ZO-1 expression analysis in IBD in vitro models.**

**A.** Protein-protein interaction network for Homo sapiens ZO-1 (also known as TJP1) was obtained using STRING with full subnetwork type. These networks are clustered through k-means clustering (number of clusters = 4) with a high confidence analysis (STRING interaction score ≥ 0.980). Each cluster is depicted with a different color. The solid and the dotted lines indicate connection within the same and different cluster respectively. Different color indicates different type of interactions are shown in the figure. **B.** ZO-1 gene expression levels based on microarray data were compared between healthy tissue (n=142), as well as non inflamed (n=30) and inflamed (n=27) tissue from UC patients. Data are shown by using a scatter plot, with each dot representing a single patient; values are shown in log2 scale and expressed as mean ± SD. Statistical analysis was performed with Kruskal-Wallis test followed by Dunn's multiple comparisons test with p-values < 0.05 were considered statistically significant; ***p < 0.001; ns = non-significance. **C-D.** Analysis of ZO-1 expression in differentiated Caco-2 cells treated with 10 ng/mL LPS + 10 ng/mL IFNγ for 24h or 48h. **C.** IF images of ZO-1 in unstimulated and 24h 10 ng/mL LPS+10 ng/mL IFNγ stimulated IBD model. White arrows indicate altered epithelial architecture. Scale bars 20 μm. **D.** ZO-1 protein levels were analyzed through WESTERN BLOT and normalized to β-tubulin expression. Each value represents the mean ± SEM n=3 independent experiments. Statistical analysis was performed with one-way ANOVA followed by Dunnett’s multiple comparison test, with *p < 0.05; **p < 0.01. **E.** IF images of Occludin in unstimulated and 24h 10 ng/mL LPS+10 ng/mL IFNγ stimulated IBD model. Scale bars 20 μm. **F.** Analysis of ZO-1 expression in differentiated Caco-2 cells silenced with siRNA GILZ. Results are shown as ZO-1/β-actin ratio. Each value represents the mean ± SEM n = 3 independent experiments. Statistical analysis was performed with Student’s t-test, with *p < 0.05.

**
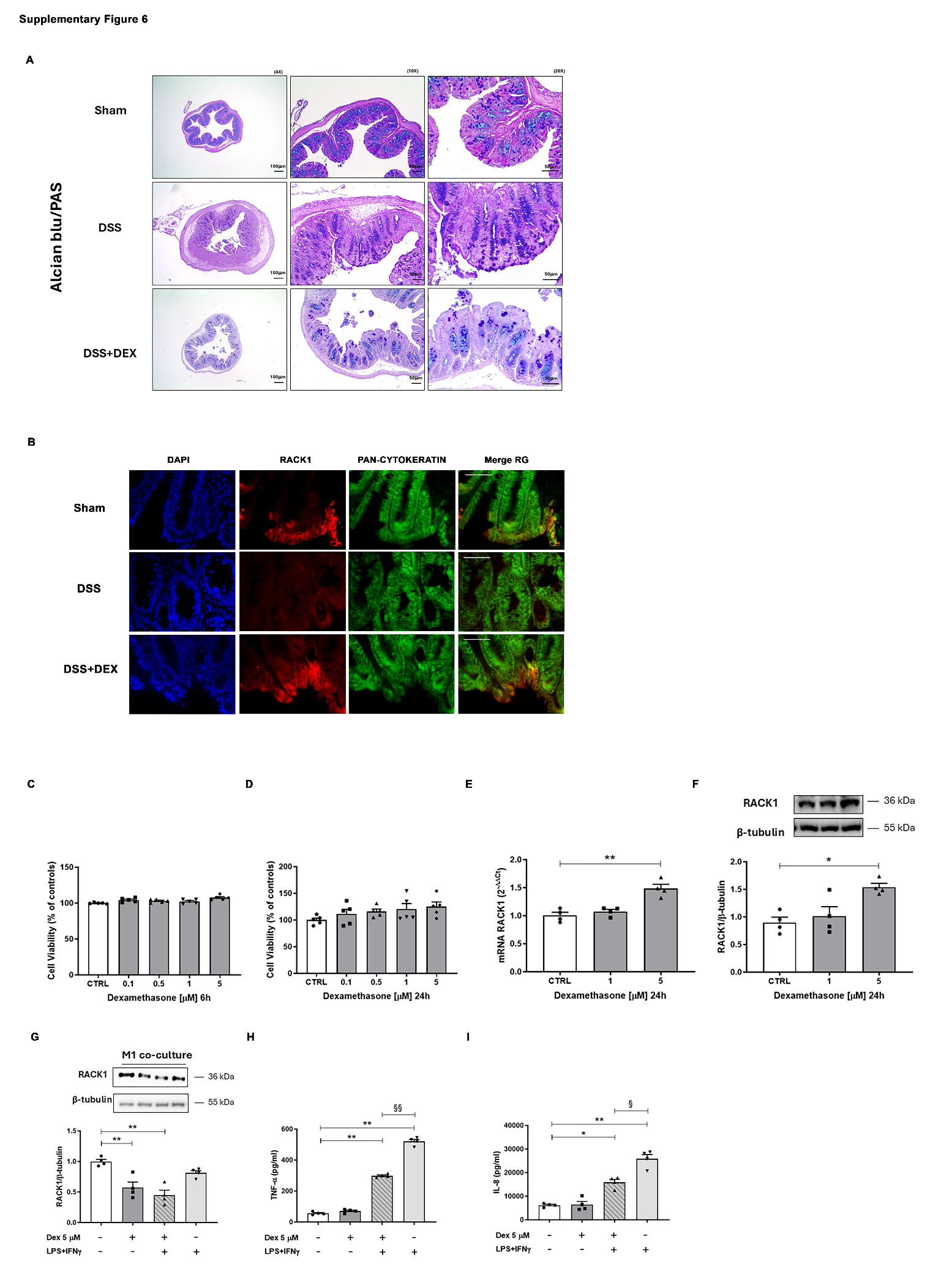
**

**Fig. S6. Characterisation of dexamethasone effect on RACK1 expression.**

**A.** Alcian Blue-P.A.S. staining of distal colons of Sham, DSS and DSS treated mice with 1mg/kg of dexamethasone (DSS+DEX). Scale bars are indicated in the figures. **B.** IF images of DAPI, RACK1 and Pan-cytokeratin in distal colon sections of Sham, DSS and DSS+DEX. Merge RG indicates merge with RACK1 (Red) and Pan-cytokeratin (Green). Scale bars 50 μm. **C, D.** Dexamethasone (Dex) treatment does not affect Caco-2 cells viability. MTT assay was performed to evaluate cell viability of Caco-2 cells treated with increasing concentrations (0.1-5 µM) of DEX or DMSO < 0.1% for vehicle controls (CTRL) for 6h (C) or 24h (D). **E, F**. Dex pharmacological treatment (1 and 5 µM) effect on RACK1 mRNA (E) and protein (F) levels after 24h of treatment. **E.** Evaluation of RACK1 mRNA was performed by qPCR and normalised to GAPDH mRNA expression. **F.** RACK1 protein levels were analysed through Western blot and normalised to β-tubulin expression. The image is a representative Western blot. **E, F.** Each value represents the mean ± SEM n = 4 independent experiments. Statistical analysis was performed with one-way ANOVA followed by Dunnett’s multiple comparison test, with *p < 0.05 and **p < 0.01. **G.** RACK1 protein levels in M1 co-cultured macrophages were analyzed through Western blot and normalized to β-tubulin expression. The image is a representative Western blot and values are means ± SEM n= 3 independent experiments. **H, I.** Dex reduces LPS+IFNγ-induced increase of TNF-α and IL-8 release in M1 macrophages. Secretion of cytokines TNF-α (H) and IL-8 (I) was evaluated in cell-free supernatants by sandwich ELISAs. Each value represents the mean ± SEM n = 4 independent experiments. Statistical analysis was performed with one-way ANOVA followed by Tukey's multiple comparison test, with *p < 0.05 and **p <0.01 vs CTRL and §p< 0.05 and §§p <0.01 vs LPS+INFγ.

**
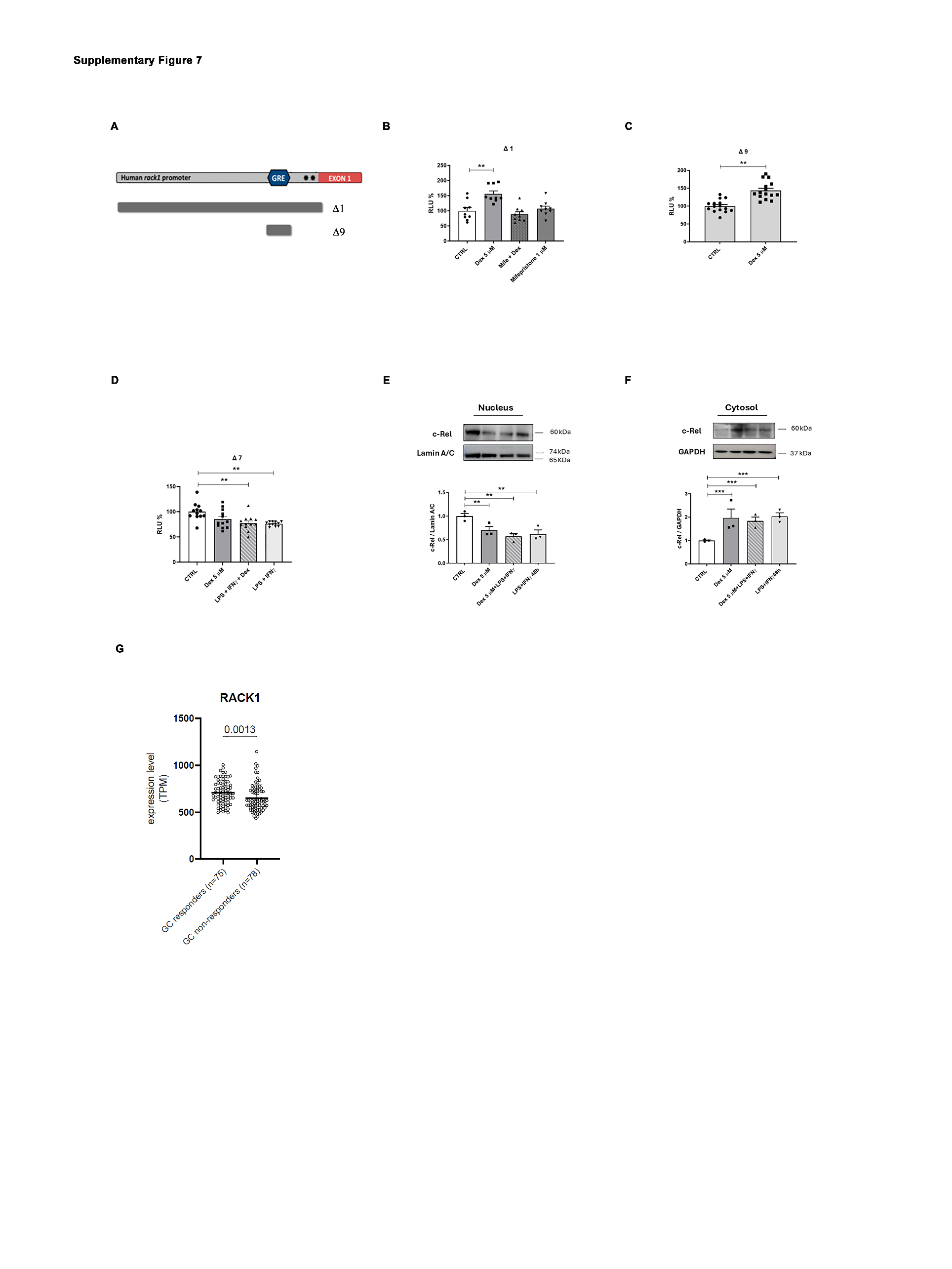
**

**Fig. S7. Dissecting dexamethasone mechanism of action on RACK1 expression**

**A.** Schematic representation of Δ1 and Δ9 RACK1 promoter deletion mutants containing GRE (Glucocorticoid Response Element) site. **B.** Differentiated Caco-2 cells (CTRL) were transiently transfected with Δ1 and subsequently treated for 24h with 5 µM Dex, 1 µM Mifepristone or pre-treated 1h with Mifepristone before adding 5 µM Dex for 24h. Luciferase activity was expressed as RLU% and compared to CTRL values assumed at 100%. Each bar represents the mean ± SEM n=3 independent experiments, in triplicate. Statistical analysis was performed with Dunnett’s test, with *p < 0.05 and **p < 0.01. **C.** Differentiated Caco-2 cells (CTRL) were transiently transfected with Δ9 and subsequently treated for 24h with 5 µM Dex. Luciferase activity was expressed as RLU% and compared to CTRL values assumed at 100%. Each bar represents the mean ± SEM n=5 independent experiments, in triplicate. Statistical analysis was performed with Student’s t-test, with **p < 0.01. **D.** Differentiated Caco-2 cells (CTRL) were transiently transfected with Δ7 and treated for 24h with 5 µM Dex, 10 ng/mL LPS + 10 ng/mL IFNγ or pre-treated 24h with LPS+IFNγ before adding 5 µM Dex for 24h. Luciferase activity was expressed as RLU% and compared to CTRL values assumed at 100%. Each bar represents the mean ± SEM n=4 independent experiments, in triplicate. Statistical analysis was performed with Dunnett’s test, with **p < 0.01. **E-F.** Differentiated Caco-2 cells (CTRL) were treated for 24h with 5 µM Dex, with 10 ng/mL LPS + 10 ng/mL IFNγ for 24h or pre-treated 24h with LPS+IFNγ before adding 5 µM Dex for 24h. Analysis of c-Rel expression in nucleus (E) and cytosol (F) fractions of differentiated Caco-2 cells. The images are representative Western blots and results are shown as c-Rel/ lamin A/C ratio for the nuclear fraction (E) and as c-Rel/GAPDH ratio for the cytosolic fractions (F). Each value represents the mean ± SEM n=3 independent experiments. Statistical analysis was performed with one-way ANOVA followed by Dunnett’s multiple comparison test, with **p < 0.01; ***p < 0.001. **G.** RACK1 gene expression levels based on GSE109142 dataset were compared between GCs responders (n=75) and GCs non-responders (n=78). Data are shown by using a scatter plot, with each dot representing a single patient. Statistical analysis was performed with the Mann-Whitney test with p-values <0.05 were considered statistically significant.

**SUPPORTING INFORMATION TEXT FOR MATERIALS AND METHODS**

**Cell cultures and treatments**

The human colorectal adenocarcinoma Caco-2 cell line was obtained from American Type Culture Collection (ATCC TIB-202; Manassas, VA, USA) and cultured in DMEM-High Glucose containing 2 mM L-glutamine, 0.1 mg/mL streptomycin, 100 IU/mL penicillin, supplemented with 10% heat-inactivated fetal bovine serum (FBS) and 1% non-essential amino acids (NEAA). According to literature data, Caco-2 cells were cultured for 21 days in complete DMEM-High Glucose medium to allow differentiation into a heterogenous mixture of intestinal epithelial cells ^1^. Medium was changed every second day.

Human THP-1 cells were purchased from the European Collection of Authenticated Cell Cultures (ECACC, Salisbury, UK) and diluted to 106 cells/mL in RPMI 1640 medium supplemented with 10% heat-inactivated FBS, 2 mM glutamine, 0.1 mg/mL streptomycin, 100 IU/mL penicillin, and 0.05 mM 2-mercaptoethanol.

THP1-Blue™ NF-kB Cells (Invivogen, UK) specifically used for 3D co-culture model were maintained in RPMI 1640 media (Thermo Fisher Scientific) supplemented with 10% heat-inactivated FBS, 0.1 mg/mL streptomycin, 100 IU/mL penicillin, 2 mM L-glutamine and 1% NEAA. THP1-Blue™ NF-kB Cells were derived from the THP-1 monocyte cell line by stable integration of an NF-kB inducible secreted alkaline phosphatase (SEAP) reporter construct that allows monitoring of NF-kB activation by determining SEAP activity. Maintenance medium was supplemented with 10 μg/mL blasticidin (Invivogen, UK) every alternate passage to maintain selection pressure.

Human primary fibroblasts were purchased from ThermoFisher Scientific, (Loughborough, UK) and were maintained in complete DMEM supplemented with 10% FBS, 0.1 mg/mL streptomycin, 100 IU/mL penicillin, 2 mM L-glutamine, and 1% NEAA.

All of the cell cultures were maintained at 37°C in 5% CO2 incubator.

BAY 11-7085, dexamethasone, and mifepristone were dissolved in DMSO (Sigma Aldrich Italia, Cas N° 67-68-5, purity 99.9%) at concentration of 10 mM and frozen at - 20°C in stock aliquots. Stocks were diluted at final concentrations in culture media at the time of use (final concentration of DMSO in culture medium < 0.1%). Control cells were treated with the same amount of DMSO. BAY 11-7085 treatment was performed for 24h at the concentration of 10 μM according to literature data (Scaife et al., 2002). Dexamethasone treatment was performed at different concentrations (0.1, 0.5, 1 and 5 µM) or DMSO for vehicle controls (CTRL) for 6 h and 24h. These concentrations were selected based on previous data ^2, 3^ as physiological (0.1 and 0.5 µM) and pharmacological (1 and 5 µM) glucocorticoid concentrations adopted for Caco-2 cells ^4, 5^. Mifepristone treatment was performed at the concentration of 1 μM according to literature data ^6^.

***In vitro* 2D and 3D co-culture model mimicking healthy and diseased state of the human intestine**

The 2D co-culture model was developed according to literature data ^7^ and is briefly summarized. Caco-2 cells were seeded on trans-well inserts (0.4 μm membrane pore size; Corning) at a density of 200.000 cells/trans-well in 12-wells plate and maintained for 21 days, monitoring the barrier integrity by Trans-Epithelial Electrical Resistance (TEER). Medium was changed every second day both in the apical and basal compartments. On day 21, IFN-γ priming of Caco-2 cells was performed whereas THP-1 cells were seeded in 75 cm2 flasks and differentiated with 100 nM PMA for 24h. Subsequently, cells were detached with Accutase® (Sigma Aldrich, St Louis, MO, USA) solution and then seeded in a 12-well plates at a density of 200.000 cells/well and allowed to attach for 1.5 h. Briefly, to induce a sufficiently pronounced barrier disruption, PMA-differentiated THP-1 cells were pre-stimulated with LPS and IFN-γ at the concentration of 10 ng/mL for 4 hours before IFN-γ-primed Caco-2 cell trans-wells were added to the 12-well plates in order to initiate the co-culture mimicking diseased state of the human intestine.

The 3D co-culture model was developed according to literature data ^8, 9^. To summarize, *in vitro* IBD mucosal models were generated using 12-well Alvetex^®^ scaffold inserts (Reprocell Europe Ltd, UK), following manufacturer instructions before cell seeding. Firstly, 500.000/insert primary fibroblasts were seeded onto the Alvetex^®^ Scaffold and cultured for 12 days in complete DMEM supplemented with 5 ng/mL TGF-β1 (Peprotech, London, UK) and 100 mg/mL ascorbic acid (Sigma Aldrich, UK); medium was replaced every 3-4 days. On day 12, 500.000/insert PMA-differentiated THP1-Blue™ NF-kB cells were seeded onto the fibroblast tissue equivalent and cultured in complete DMEM for a further 2 days. Caco-2 cells were then added to the 3D cultures on day 14 at a density of 400.000 cells/insert and were cultured in complete DMEM for an additional 21 days, during which the medium was replaced every 3-4 days. After 21 days, the IBD models were treated with 10 ng/mL LPS+IFNγ for 24h and the barrier integrity was monitored by Trans-Epithelial Electrical Resistance (TEER).

**Monitoring of** **barrier integrity by Trans-Epithelial Electrical Resistance (TEER)**

TEER was measured using an Ohm-meter to assess the barrier development of the Caco-2 cell layer and monitor its integrity in the presence of inflammatory stimuli ^7, 10, 11^. Results were corrected for the blank and multiplied by the filter size (1.12 cm2) to obtain the final results in Ohm per cm2 (Ω·cm2) in 2D co-culture ^11^ whereas in 3D model, final TEER values were determined by subtraction of the blank insert TEER value and multiplying by cell-surface area of the Alvetex^®^ scaffold ^9^.

***In vitro* 3D model paraffin embedding and hematoxylin and eosin (H&E) staining**

*In vitro* 3D models were washed in PBS 1X prior to fixation in 4% paraformaldehyde for 2 h at room temperature. Samples were dehydrated through a series of ethanols, followed by incubation in Histoclear (National Diagnostics, United States) then in 1:1 Histoclear:wax ^8, 9^. Then, models were further incubated in wax before embedding and sectioning. Paraffin sections were deparaffinized in Histoclear and rehydrated to distilled H2O (d H2O) before being stained in Mayer’s Hematoxylin (Sigma-Aldrich) for 5 minutes. Slides were then washed in dH_2_O and submerged in alkaline ethanol to blue the nuclei. Samples were dehydrated to 95% ethanol counter-stained in Eosin followed by dehydration to 100% ethanol. Slides were cleared twice in Histoclear and mounted in Omni-mount (National Diagnostics) before imaging on a Leica microscope.

**Datasets, gene expression analysis and STRING**

Expression data from the Gene Expression Omnibus (GEO) database of whole human genome arrays and the ArrayExpress Archive of Functional Genomics Data (ArrayExpress) ^12, 13^, generated using the Affymetrix Human Genome-U133-Plus-2.0 platform, were downloaded and processed through the Genevestigator V3 suite (NEBION AG, Zurich, Switzerland) ^14^. The microarray data in Genevestigator were normalized at two levels: robust multiarray average within experiments (using the Bioconductor package "affy" and a customized version of the package "affyExtensions") and trimmed mean adjustment to a target for normalization between datasets. Regarding the latter, the trimmed mean is calculated by determining the mean of all expression values in an experiment (across all samples) after excluding the top 5% and the bottom 5%. The combination of these two levels of normalization ensures high comparability of the data across different experiments, enabling pooling of data without additional normalization.

The Genevestigator database was queried in September 2023. We included in the analysis only arrays for mRNA samples that (1) were not obtained by laser capture microdissection and (2) were not subjected to *in vitro* experimental treatments. Normalized gene expression data (expressed as log2 values) were downloaded from 528 arrays of healthy and diseased tissue. Specifically, were obtained samples from the gastrointestinal (GI) tracts of healthy subjects (n = 276 from datasets GSE3526, GSE7307, GSE18105, GSE23878, GSE10714, GSE10191, GSE9686, GSE38713, GSE8671, GSE13911, GSE20916, GSE19826, GSE4183, GSE28177, E-MEXP-1828, E-MEXP-1823, GSE26886, and GSE43346), from the gut of ulcerative colitis (UC) patients (non-inflamed tissue: n = 30 from datasets GSE13367, GSE11831, GSE9452, E-TABM-118, and E-MEXP-2083; inflamed tissue: n = 27 from datasets GSE13367, GSE11831, GSE9452, E-TABM-118, and E-MEXP-2083) and CD patients (non-inflamed tissue: n = 29 from datasets GSE11831, GSE9452, E-TABM-118, and E-MEXP-1225; inflamed tissue: n = 24, from datasets GSE11831, GSE9452, E-TABM-118, E-MEXP-2083, and E-MEXP-1225). Bulk RNAseq from the GSE109142 dataset, described in ref. 15, was used to evaluate the expression of RACK1.

“STRING: functional protein association networks” database (<https://string-db.org/cgi/input?sessionId=didPoYPnXmcb&input_page_show_search=on>) was used to investigate the interactions involving SRC and two of its binding partners, RACK1 and ZO-1. We queried STRING to identify known and predicted protein-protein interactions specifically involving SRC, RACK1, and ZO-1, utilizing data from experimental repositories, computational predictions, and literature mining. Subsequently, we visualized and analyzed the resulting protein networks using network analysis tools to gain insights into the functional relationships and biological processes involving SRC, RACK1, and ZO-1. This approach enabled us to explore the specific interactions of SRC with RACK1 and ZO-1 and provided valuable context for understanding the molecular mechanisms underlying these interactions in cellular signaling pathways.

**Plasmid DNA Preparation, Transient Transfections, and Luciferase Assays**

The Δ1, Δ7, Δ9 and Δ11 reporter plasmid constructs were purified with the HiSpeed® Plasmid Midi Kit (Qiagen, Valencia, CA, United States) and then DNA was quantified and assayed for purity using Quantus™ fluorometer (Promega, Madison, WI, United States). Plasmids transient transfection was carried out using Lipofectamine® 2000 (Invitrogen Carlsbad, CA, United States), following the manufacturer's instructions. Each luciferase reporter construct plasmid DNA was co-transfected with the pRL-TK renilla luciferase expressing vector to measure the transfection efficiency (Promega, Madison, WI, United States). Differentiated Caco-2 cells were treated as described in figure legends and then lysed with Passive Lysis Buffer 1X, provided by the Dual-Luciferase Reporter Assay System (Promega, Madison, WI, United States), following the manufacturer specifications. Luminescence was measured with a 20/20n Luminometer (Turner Bio-Systems, Sunnyvale, CA, United States), with 10s integration time.

**References**

1. Ferraretto A, Gravaghi C, Donetti E, Cosentino S, Donida BM, Bedoni M, Lombardi G, Fiorilli A, Tettamanti G. New methodological approach to induce a differentiation phenotype in Caco-2 cells prior to post-confluence stage. Anticancer Res. 2007 Nov-Dec;27(6B):3919-25.
2. Del Vecchio I, Zuccotti A, Pisano F, Canneva F, Lenzken SC, Rousset F, Corsini E, Govoni S, Racchi M. Functional mapping of the promoter region of the GNB2L1 human gene coding for RACK1 scaffold protein. Gene. 2009 Feb 1;430(1-2):17-29. doi: 10.1016/j.gene.2008.10.005.
3. Buoso E, Lanni C, Molteni E, Rousset F, Corsini E, Racchi M. Opposing effects of cortisol and dehydroepiandrosterone on the expression of the receptor for Activated C Kinase 1: implications in immunosenescence. Exp Gerontol. 2011 Nov;46(11):877-83. doi: 10.1016/j.exger.2011.07.007.
4. Cavicchi M, Whittle BJ. Regulation of induction of nitric oxide synthase and the inhibitory actions of dexamethasone in the human intestinal epithelial cell line, Caco-2: influence of cell differentiation. Br J Pharmacol. 1999 Oct;128(3):705-15. doi: 10.1038/sj.bjp.0702827. PMID: 10516652; PMCID: PMC1571669.
5. Raddatz D, Toth S, Schwörer H, Ramadori G. Glucocorticoid receptor signaling in the intestinal epithelial cell lines IEC-6 and Caco-2: evidence of inhibition by interleukin-1beta. Int J Colorectal Dis. 2001 Nov;16(6):377-83. doi: 10.1007/s003840100331. PMID: 11760899.
6. Wang D, Zhang H, Lang F, Yun CC. Acute activation of NHE3 by dexamethasone correlates with activation of SGK1 and requires a functional glucocorticoid receptor. Am J Physiol Cell Physiol. 2007 Jan;292(1):C396-404. doi: 10.1152/ajpcell.00345.2006. Epub 2006 Sep 13. PMID: 16971495; PMCID: PMC2695591.
7. Kämpfer AAM, Urbán P, Gioria S, Kanase N, Stone V, Kinsner-Ovaskainen A. Development of an in vitro co-culture model to mimic the human intestine in healthy and diseased state. Toxicol In Vitro. 2017 Dec;45(Pt 1):31-43. doi: 10.1016/j.tiv.2017.08.011.
8. Darling NJ, Mobbs CL, González-Hau AL, Freer M, Przyborski S. Bioengineering Novel in vitro Co-culture Models That Represent the Human Intestinal Mucosa With Improved Caco-2 Structure and Barrier Function. Front Bioeng Biotechnol. 2020 Aug 31;8:992. doi: 10.3389/fbioe.2020.00992. PMID: 32984279; PMCID: PMC7487342.
9. Mobbs CL, Darling NJ, Przyborski S. An in vitro model to study immune activation, epithelial disruption and stromal remodelling in inflammatory bowel disease and fistulising Crohn's disease. Front Immunol. 2024 Feb 12;15:1357690. doi: 10.3389/fimmu.2024.1357690. PMID: 38410518; PMCID: PMC10894943.
10. Cheng ZF, Pai RK, Cartwright CA. Rack1 function in intestinal epithelia: regulating crypt cell proliferation and regeneration and promoting differentiation and apoptosis. Am J Physiol Gastrointest Liver Physiol. 2018 Jan 1;314(1):G1-G13. doi: 10.1152/ajpgi.00240.2017.
11. Cheng ZF, Cartwright CA. Rack1 maintains intestinal homeostasis by protecting the integrity of the epithelial barrier. Am J Physiol Gastrointest Liver Physiol. 2018 Feb 1;314(2):G263-G274. doi: 10.1152/ajpgi.00241.2017.
12. Edgar R, Domrachev M, Lash AE. Gene Expression Omnibus: NCBI gene expression and hybridization array data repository. Nucleic Acids Res. 2002 Jan 1;30(1):207-10. doi: 10.1093/nar/30.1.207. PMID: 11752295; PMCID: PMC99122.
13. Kolesnikov N, Hastings E, Keays M, Melnichuk O, Tang YA, Williams E, Dylag M, Kurbatova N, Brandizi M, Burdett T, Megy K, Pilicheva E, Rustici G, Tikhonov A, Parkinson H, Petryszak R, Sarkans U, Brazma A. ArrayExpress update--simplifying data submissions. Nucleic Acids Res. 2015 Jan;43(Database issue):D1113-6. doi: 10.1093/nar/gku1057. Epub 2014 Oct 31. PMID: 25361974; PMCID: PMC4383899.
14. Hruz T, Laule O, Szabo G, Wessendorp F, Bleuler S, Oertle L, Widmayer P, Gruissem W, Zimmermann P. Genevestigator v3: a reference expression database for the meta-analysis of transcriptomes. Adv Bioinformatics. 2008;2008:420747. doi: 10.1155/2008/420747. Epub 2008 Jul 8. PMID: 19956698; PMCID: PMC2777001.
15. Haberman Y, Karns R, Dexheimer PJ, Schirmer M, Somekh J, Jurickova I, Braun T, Novak E, Bauman L, Collins MH, Mo A, Rosen MJ, Bonkowski E, Gotman N, Marquis A, Nistel M, Rufo PA, Baker SS, Sauer CG, Markowitz J, Pfefferkorn MD, Rosh JR, Boyle BM, Mack DR, Baldassano RN, Shah S, Leleiko NS, Heyman MB, Grifiths AM, Patel AS, Noe JD, Aronow BJ, Kugathasan S, Walters TD, Gibson G, Thomas SD, Mollen K, Shen-Orr S, Huttenhower C, Xavier RJ, Hyams JS, Denson LA. Ulcerative colitis mucosal transcriptomes reveal mitochondriopathy and personalized mechanisms underlying disease severity and treatment response. Nat Commun. 2019 Jan 3;10(1):38. doi: 10.1038/s41467-018-07841-3. PMID: 30604764; PMCID: PMC6318335.
